# Supplementary material for: ChromTime: modeling spatio-temporal dynamics of chromatin marks
Source: Genome Biol. 2018 Aug 10;19:109. doi: 10.1186/s13059-018-1485-2 (PMC6085762; doi:10.1186/s13059-018-1485-2)
Supplement: Supplementary file 1 — Additional figures supporting the main analyses. (PDF 8541 kb) [file 13059_2018_1485_MOESM1_ESM.pdf]

Fig S1

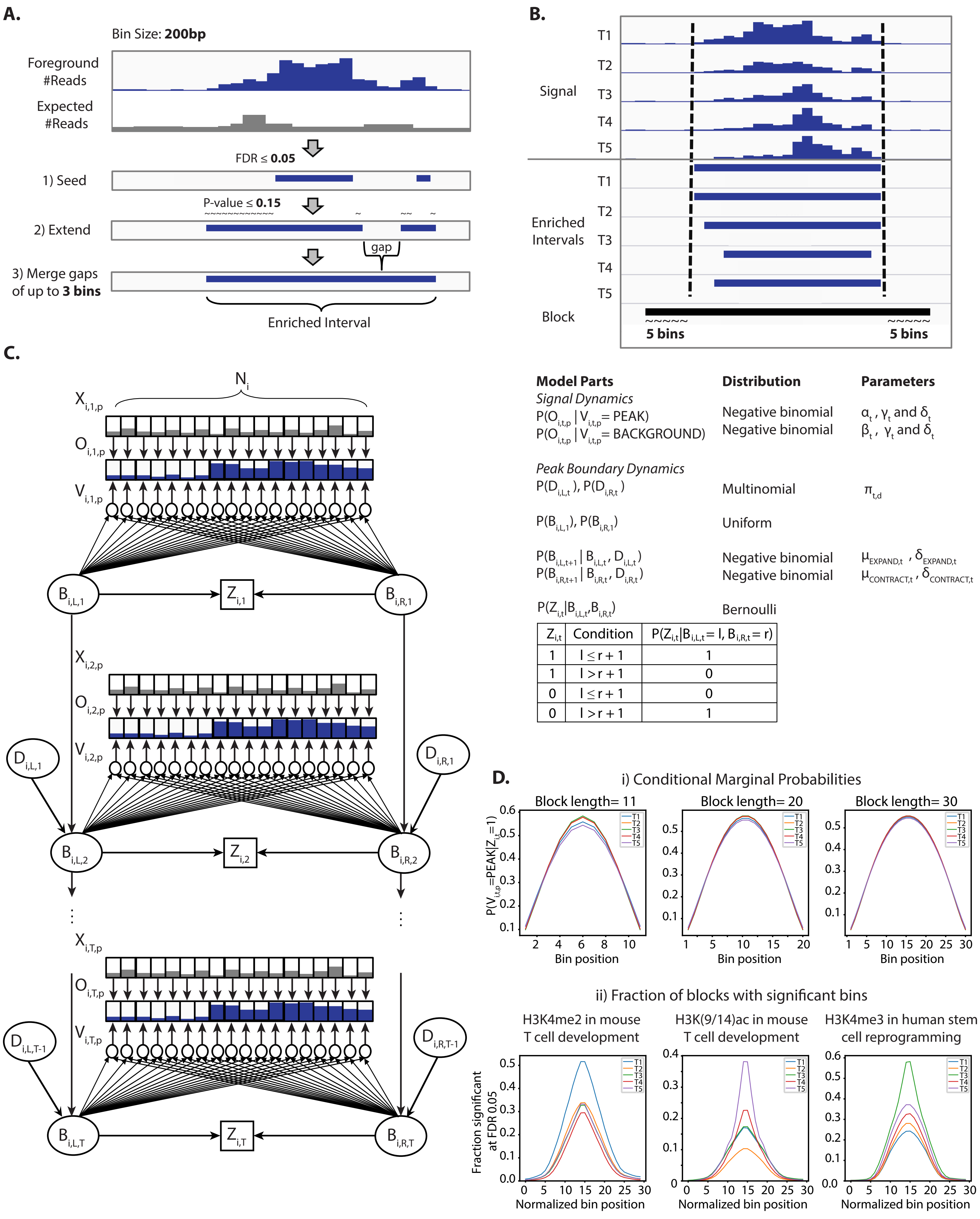

A.
Bin Size: 200bp
Foreground #Reads
Expected #Reads
FDR ≤ 0.05
1) Seed
P-value ≤ 0.15
2) Extend
gap
3) Merge gaps of up to 3 bins
Enriched Interval
B.
Signal
T1
T2
T3
T4
T5
Enriched Intervals
Block
5 bins
5 bins
C.
Model Parts
Distribution
Parameters
Signal Dynamics
$P(O_{i,t,p} | V_{i,t,p} = \text{PEAK})$ Negative binomial α_t, γ_t and δ_t
$P(O_{i,t,p} | V_{i,t,p} = \text{BACKGROUND})$ Negative binomial β_t, γ_t and δ_t
Peak Boundary Dynamics
$P(D_{i,L,t})$, $P(D_{i,R,t})$ Multinomial $\pi_{t,d}$
$P(B_{i,L,1})$, $P(B_{i,R,1})$ Uniform
$P(B_{i,L,t+1} | B_{i,L,t}, D_{i,L,t})$ Negative binomial $\mu_{EXPAND,t}$, $\delta_{EXPAND,t}$
$P(B_{i,R,t+1} | B_{i,R,t}, D_{i,R,t})$ Negative binomial $\mu_{CONTRACT,t}$, $\delta_{CONTRACT,t}$
$P(Z_{i,t} | B_{i,L,t}, B_{i,R,t})$ Bernoulli
$Z_{i,t}$ | Condition | $P(Z_{i,t} | B_{i,L,t} = l, B_{i,R,t} = r)$
1 | $l \le r+1$ | 1
1 | $l > r+1$ | 0
0 | $l \le r+1$ | 0
0 | $l > r+1$ | 1
N_i
$X_{i,1,p}$
$O_{i,1,p}$
$V_{i,1,p}$
$B_{i,L,1}$
$Z_{i,1}$
$B_{i,R,1}$
$X_{i,2,p}$
$O_{i,2,p}$
$V_{i,2,p}$
$D_{i,L,1}$
$D_{i,R,1}$
$B_{i,L,2}$
$Z_{i,2}$
$B_{i,R,2}$
$X_{i,T,p}$
$O_{i,T,p}$
$V_{i,T,p}$
$D_{i,L,T-1}$
$D_{i,R,T-1}$
$B_{i,L,T}$
$Z_{i,T}$
$B_{i,R,T}$
D.
i) Conditional Marginal Probabilities
Block length= 11
Block length= 20
Block length= 30
$P(V_{i,t,p}=\text{PEAK} | Z_{i,t}=1)$
Bin position
ii) Fraction of blocks with significant bins
H3K4me2 in mouse T cell development
H3K(9/14)ac in mouse T cell development
H3K4me3 in human stem cell reprogramming
Fraction significant at FDR 0.05
Normalized bin position

Fig S1: Details of the ChromTime method. (A) Calling enriched intervals at a given time point during the block finding stage of ChromTime. A schematic of the number of foreground (blue bars) and expected (grey bars) reads are shown for each bin within a genomic region. Significantly enriched bins are called after correcting for multiple testing across the genome at FDR of 0.05 (seed step). Enriched bins are extended locally in both directions until a bin is found whose enrichment is not significant at a P-value of 0.15 (extend step). Extended bins are marked with "~" signs in the schematic. Continuous intervals are joined if they are separated by gaps of up to MIN_GAP bins (3 by default). **(B)** Enriched intervals from (**A**) that overlap across time are grouped into blocks. Blocks are further extended by BLOCK_EXTEND bins (5 by default, or up to the midpoint to their nearest neighbor block) up and down-stream from the left-most and the right-most position respectively of the enriched intervals within blocks. **(C)** Graphical model for the ChromTime mixture model for block i with length N_i bins in a dataset with T time points. The model has T levels of $B_{i,L,t}, B_{i,R,t}, V_{i,t,p}, O_{i,t,p}$ and $X_{i,t,p}$ variables – one for each time point. At each time point, the total number of $V_{i,t,p}, O_{i,t,p}$ and $X_{i,t,p}$ variables is N_i. In addition, there are $T-1$ levels of $D_{i,L,t}$ and $D_{i,R,t}$ variables – one level for each pair of consecutive time points. Observed and latent variables are represented as boxes and circles, respectively. Example values for observed and expected read counts for each bin within the block are represented with blue and grey bars, respectively, inside each box. All conditional and prior probabilities from the model, their distribution type and their parameters estimated during the expectation maximization phase, are listed on the right. The probability mass function for $P(Z_{i,t}|B_{i,L,t}, B_{i,R,t})$ is given below all parameters. **(D)** (**i**) The conditional probabilities $P(V_{i,t,p} = \text{PEAK}|Z_{i,t} = 1)$, which model the probability that a bin at position p at time point t in block i is annotated as PEAK, conditioned on the requirement that the left end boundary is placed before the right end boundary, are plotted as a function of the bin position p for each time point. The values of $P(V_{i,t,p} = \text{PEAK}|Z_{i,t} = 1)$ are shown for blocks of length 11 bins (minimum block length in ChromTime in the absence of adjacent blocks within 5 bins (default for BLOCK_EXTEND)), 20 bins and 30 bins (default value of the MAX_BINS parameter, Additional file 2: **Supplementary Methods**) in a dataset with 5 time points. The values are computed after marginalizing out the observed read counts and all latent variables, except for $V_{i,t,p}$, in the joint probability of all variables in the model (**Methods**). Lines are largely overlapping and have their maximum at the center bin. (**ii**) The average fraction of blocks with significant bins at FDR 0.05 from the seed step during the block finding stage of ChromTime as a function of the bin position for three time courses (left to right): H3K4me2 and H3K(9/14)ac in mouse T cell development[1] and H3K4me3 in human stem cell reprogramming[2]. The average fraction is shown for blocks of length up to 30 bins (longer blocks are excluded, because by default they are split in halves for computational reasons). Each line corresponds to one time point. X-axis corresponds to bin positions within blocks when blocks are rescaled uniformly to length of 30 bins. In all three cases, the average fraction of blocks with significant bins has its maximum at the central positions of the blocks, which is consistent with the assumption of the method that the conditional probability $P(V_{i,t,p} = \text{PEAK}|Z_{i,t} = 1)$ peaks at central positions.

Fig S2

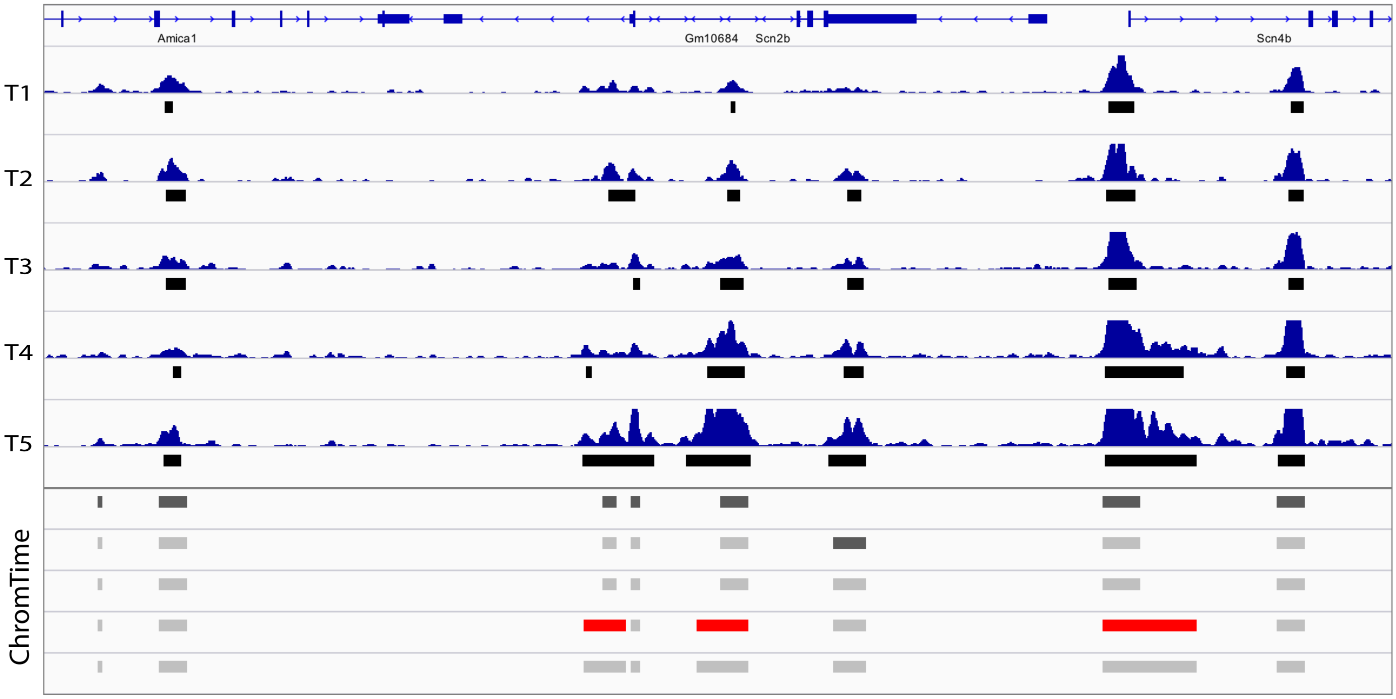

Fig S2: Sample output from ChromTime with expanding peaks. Genome browser screenshot with sample output of ChromTime for H3K4me2 from the T cell development time course in mouse[1] with 5 time points at the Gm10684/Scn2b locus. Time points 1, 2 and 3 correspond to in vitro differentiated T cell precursors (FLDN1, FLDN2a, and FLDN2b), whereas time points 4 and 5 correspond to in vivo purified thymocytes (ThyDN3 and ThyDP). The input ChIP-seq signal and MACS2[3] peaks (black boxes under each signal track) are shown in the upper panel of the screenshot. The predicted ChromTime peaks colored by their boundary dynamics for each block at each time point are shown in the bottom panel. The first peak in each block is colored in dark grey. Each subsequent peak is colored with respect to the predicted dynamic relative to its previous time point. Peaks with steady boundaries on both sides are shown in light grey, and those with at least one expanding are shown in red. Not shown in the figure are contracting peaks, peaks at single time points and peaks with opposite dynamics (Expand on the left and Contract on the right, or vice versa), which would be colored in blue, orange and black, respectively. See **Fig 2** for examples of predicted contracting peaks.

Fig S3

H3K4me2 in mouse T cell development

	Base %	C/C	C/S	S/C	S/S	C/E	E/C	E/E	E/S	S/E
C/C	1.9	4.7	1.2	1.5	-1.9	-0.6	-1.2	-3.7	-5.9	-6.7
C/S	9.5	1.3	2.1	0.7	-1.0	-1.7	-2.8	-6.5	-6.2	-4.9
S/C	8.5	1.3	0.7	2.2	-0.9	-0.7	-0.6	-6.4	-6.5	-6.5
S/S	62.3	-2.4	-1.4	-1.5	0.3	-1.9	-1.0	-2.5	-1.3	-1.4
C/E	< 0.1	-0.7	-1.1	-2.6	-0.5	0.0	0.0	-0.5	-0.2	1.7
E/C	< 0.1	-0.9	-2.8	0.8	-2.1	0.0	1.4	-0.4	-0.2	-1.1
E/E	0.4	-2.7	-5.5	-5.5	-3.4	-0.3	1.1	5.8	2.2	2.5
E/S	3.0	-6.0	-5.2	-5.9	-1.6	-0.2	0.0	2.8	3.5	2.4
S/E	2.9	-6.0	-6.1	-8.4	-1.6	0.8	-1.5	2.9	2.2	3.5
Base %		0.8	6.8	6.7	70.2	< 0.1	< 0.1	0.5	3.4	3.4

H3K(9,14)ac in mouse T cell development

	Base %	C/C	C/S	S/C	S/S	C/E	E/C	E/E	E/S	S/E
C/C	2.2	3.0	1.3	-0.2	-2.7	0.0	-0.4	-0.8	-1.9	-2.4
C/S	7.8	0.9	2.9	1.8	-0.9	0.0	-0.9	-2.4	-4.6	-4.0
S/C	6.6	2.9	1.6	3.1	-1.0	0.0	-0.8	-2.3	-4.1	-3.9
S/S	74.5	-5.1	-0.8	-0.8	0.2	0.0	-1.9	-3.0	-1.1	-1.2
C/E	0.0	0.0	0.0	0.0	0.0	0.0	0.0	0.0	0.0	0.0
E/C	< 0.1	0.0	-0.5	-0.5	-0.3	0.0	1.4	0.0	-0.2	-0.2
E/E	0.2	-0.2	-1.5	-1.6	-3.4	0.0	0.0	3.6	1.8	2.3
E/S	1.8	-1.4	-4.0	-5.0	-2.6	0.0	-0.3	1.9	4.0	3.1
S/E	1.8	-1.4	-4.9	-5.0	-2.7	0.0	-0.3	2.1	3.1	4.0
Base %		0.1	2.1	2.1	72.4	0.0	< 0.1	0.2	1.8	2.0

Base %: 0 – 100

Log2 Fold Enrichment: -6 – 0 – 6

Fig S3: Reproducibility of ChromTime predictions across biological replicates. Average $\log_2$ fold enrichments (positive values, red) and depletions (negative values, blue) of base level overlap between peaks with predicted spatial dynamics in biological replicate experiments for H3K4me2 and H3K(9/14)ac in mouse T cell development[1]. The first column and the last row in each table show the average baseline fraction of bases covered by each dynamic out of all bases covered by ChromTime peaks. The averages are taken across all pairs of consecutive time points in each time course.

Fig S4

A. i) H3K27ac in human stem cell reprogramming

Dynamic	% all block bases	H1-IMR90 shared DHSs	H1-IMR90 shared CEBPB	H1-IMR90 shared P300	H1-specific DHSs	H1-specific CEBPB	H1-specific P300	H1 NANOG	H1 OCT4	IMR90-specific DHSs	IMR90-specific CEBPB	IMR90-specific P300
T1-Tn Steady	2	3.6	3.7	1.4	0.8	1.2	1.3	1.6	1.3	0.6	1.2	1.7
Tx-Tn Expand	14	1.4	1.4	1	3	3.5	2	3.6	3.8	0.2	0.3	0.3
T1-Tx Contract	26	0.5	0.7	1	0.2	0.2	0.3	0.2	0.2	2.3	2	2.2
Base %		10	0.5	31	4.9	0.2	7.9	0.4	0.3	7.8	2.5	1.5

B. i) H3K27ac in human stem cell reprogramming

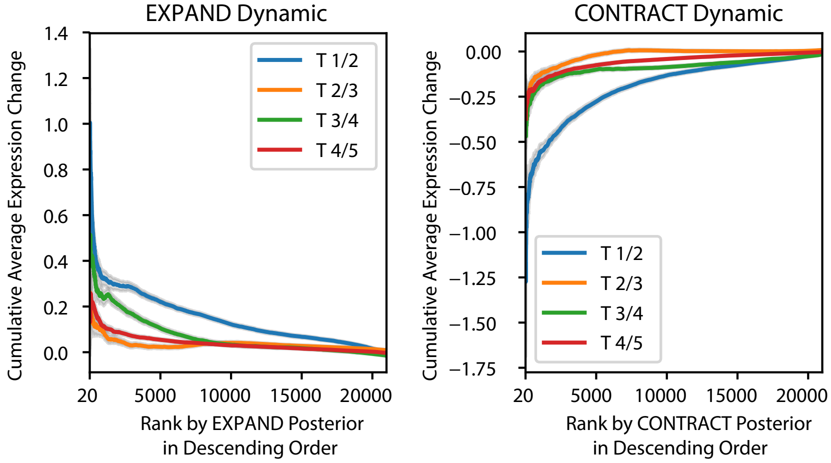

ii) H3K4me3 in human stem cell reprogramming

Dynamic	% all block bases	H1-IMR90 shared DHSs	H1-IMR90 shared CEBPB	H1-IMR90 shared POL2	H1-IMR90 shared RAD21	H1-specific DHSs	H1-specific CEBPB	H1-specific POL2	H1-specific RAD21	IMR90-specific CEBPB	IMR90-specific POL2	IMR90-specific RAD21	IMR90-specific DHSs
T1-Tn Steady	26	1.5	1.5	1.8	1.3	0.6	0.9	1.2	0.9	1.2	1.2	1.2	0.8
Tx-Tn Expand	38	0.7	0.7	0.4	0.9	1.9	1.6	1.3	1.6	0.4	0.4	0.5	0.3
T1-Tx Contract	22	1.2	1.1	1.3	1	0.3	0.4	0.5	0.4	1.9	1.9	1.8	2.4
Base %		18	0.7	4.5	1.4	11	0.3	3	0.9	1.7	4.2	1	3.1

ii) H3K4me3 in human stem cell reprogramming

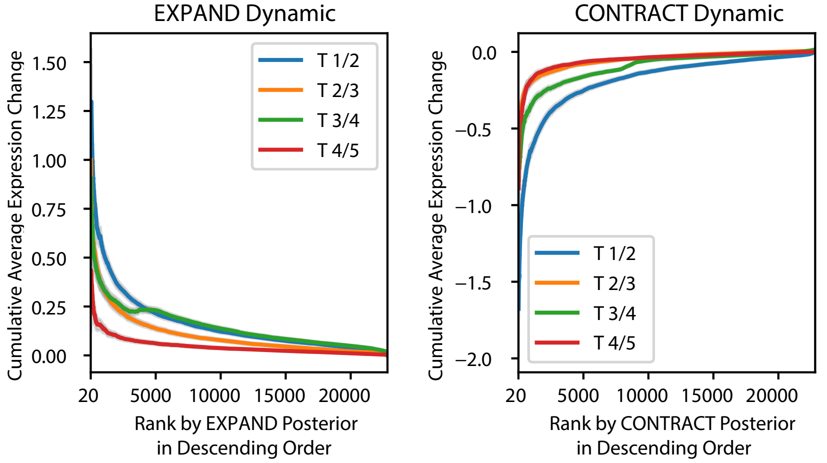

iii) ATAC-seq in mouse stem cell reprogramming

Dynamic	% all block bases	MEF-ES shared P300	MEF RUNX1	MEF specific P300	ES OCT4	ES SOX2	ES NANOG	ES ESRRB	ES specific P300
T1-Tn Steady	22	2.5	0.9	1.0	0.7	0.7	0.6	0.5	0.5
Tx-Tn Expand	33	0.6	0.1	0.1	2.2	2.2	2.3	2.1	2.4
T1-Tx Contract	38	0.5	1.9	1.9	0.1	0.1	0.1	0.3	0.1
Base %		0.2	6.7	7.7	3.4	3.8	2.6	0.3	2.5

iii) ATAC-seq in mouse stem cell reprogramming

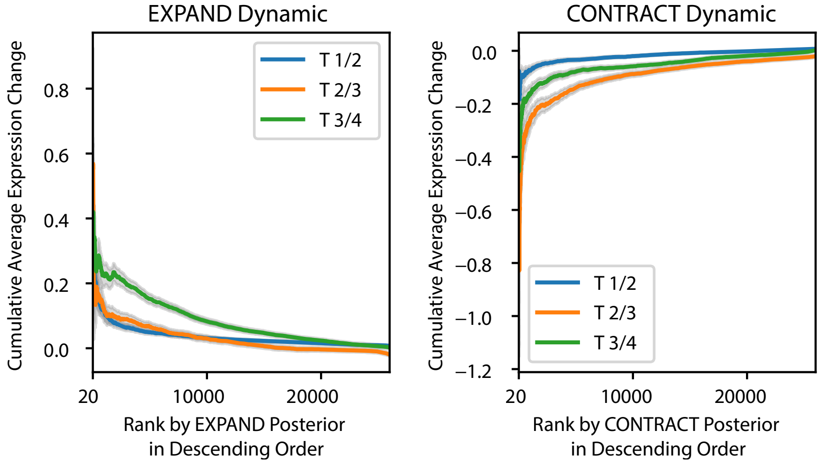

iv) DNase-seq in human fetal brain development

Dynamic	% all block bases	H1 NANOG	H1 OCT4
T1-Tn Steady	24	1.1	0.8
Tx-Tn Expand	52	0.2	0.2
T1-Tx Contract	19	3	3.4
Base %		0.8	0.5

iv) DNaseI-seq in human fetal brain development

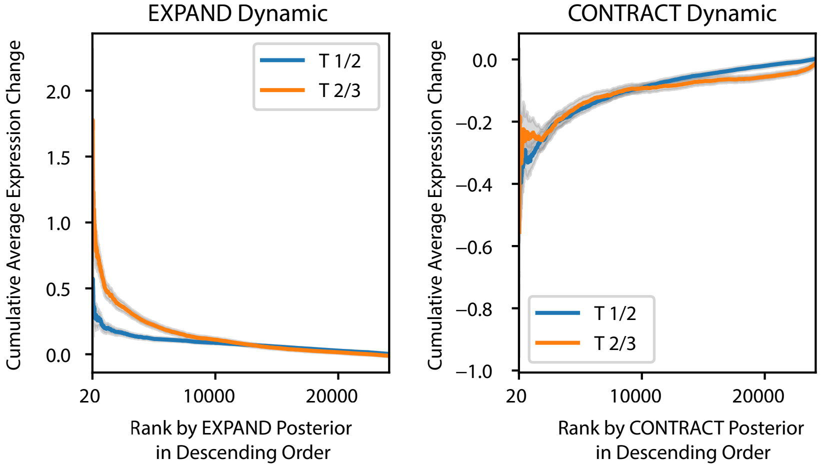

Fig S4: **Changes in TF binding, DHSs and gene expression at ChromTime predicted dynamics.** (**A**) Three sets of eaks were examined as defined in **Fig 3A**: T1-Tn Steady, Tx-Tn Expand, and T1-Tx Contract. (**i**) For predicted H3K27ac dynamics in human stem cell reprogramming[2], fold enrichments are shown for DHSs, CEBP, P300, OCT4 and NANOG. OCT4 and NANOG are key factors for maintaining pluripotency. IMR90 and H1 denote IMR90 human fetal lung fibroblast cells and H1 human embryonic stem cells, respectively, which resemble biologically the first and the last time point in this time course. **(ii)** As in **(i)** for different H3K4me3 dynamics during human stem cell reprogramming with enrichments for DHSs, CEBP, POL2 and RAD21 binding[2]. **(iii)** As in **(i-ii)** for different ATAC-seq dynamics in a mouse stem cell reprogramming time course with four time points with enrichments for RUNX1 in mouse embryonic fibroblasts (MEF), and OCT4, SOX2, NANOG, ESRRB in mouse embryonic stem cells (ES) and for shared, MEF-specific and ES-specific binding of P300 [4]. Time points correspond to MEF, 48 hours after induction of reprogramming, late intermediate pre-induced pluripotent stem cells and ES. RUNX1 is a fibroblast-specific TF, whereas OCT4, SOX2, NANOG and ESRRB are key pluripotency factors. **(iv)** as in **(i-iii)** for NANOG and OCT4 in H1 human embryonic stem cells (H1) for different DNase-seq dynamics in a human fetal brain development time course with three time points[5]. Time points correspond to H1, H1-derived neuronal progenitors and fetal brain tissue. **(B)** As in **Fig 3B**, boundaries of predicted peaks of **(i)** H3K27ac and **(ii)** H3K4me3 in human stem cell reprogramming[2], **(iii)** ATAC-seq in mouse stem cell reprogramming[4] and **(iv)** DNase-seq in human fetal brain development[5] in blocks with at least one predicted non-zero length peak overlapping annotated TSSs were sorted in decreasing order by their posterior probability for EXPAND dynamic (left plots) and CONTRACT dynamic (right plots) at each pair of consecutive time points (Additional file 2: **Supplementary Methods**). For each peak boundary, gene expression differences were calculated between consecutive time points as the average difference of all TSSs overlapping the region spanning from the left-most to the right-most coordinate of peaks within the block, with positive values corresponding to increasing expression. For each posterior rank (X-axis) the plots show the cumulative average gene expression change (Y-axis). In all datasets, expanding boundaries associated with increase of gene expression and contracting boundaries associated with decrease of gene expression. Shaded regions correspond to 95% confidence intervals.

Fig S5

A.

ChromTime SINGLE

i) H3K4me2 in mouse T cell development

ii) H3K4me3 in human stem cell reprogramming

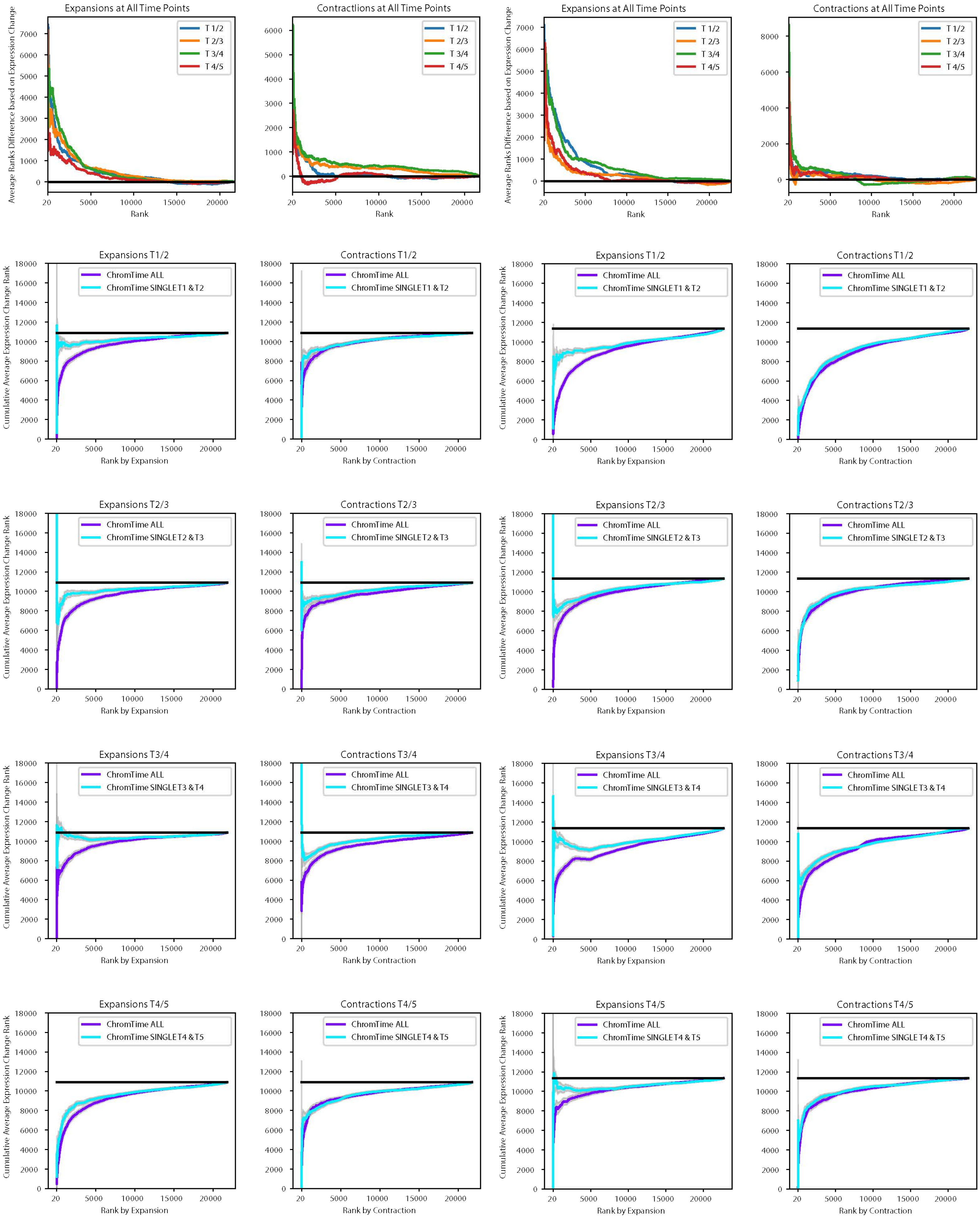

B.

MACS2

i) H3K4me2 in mouse T cell development

ii) H3K4me3 in human stem cell reprogramming

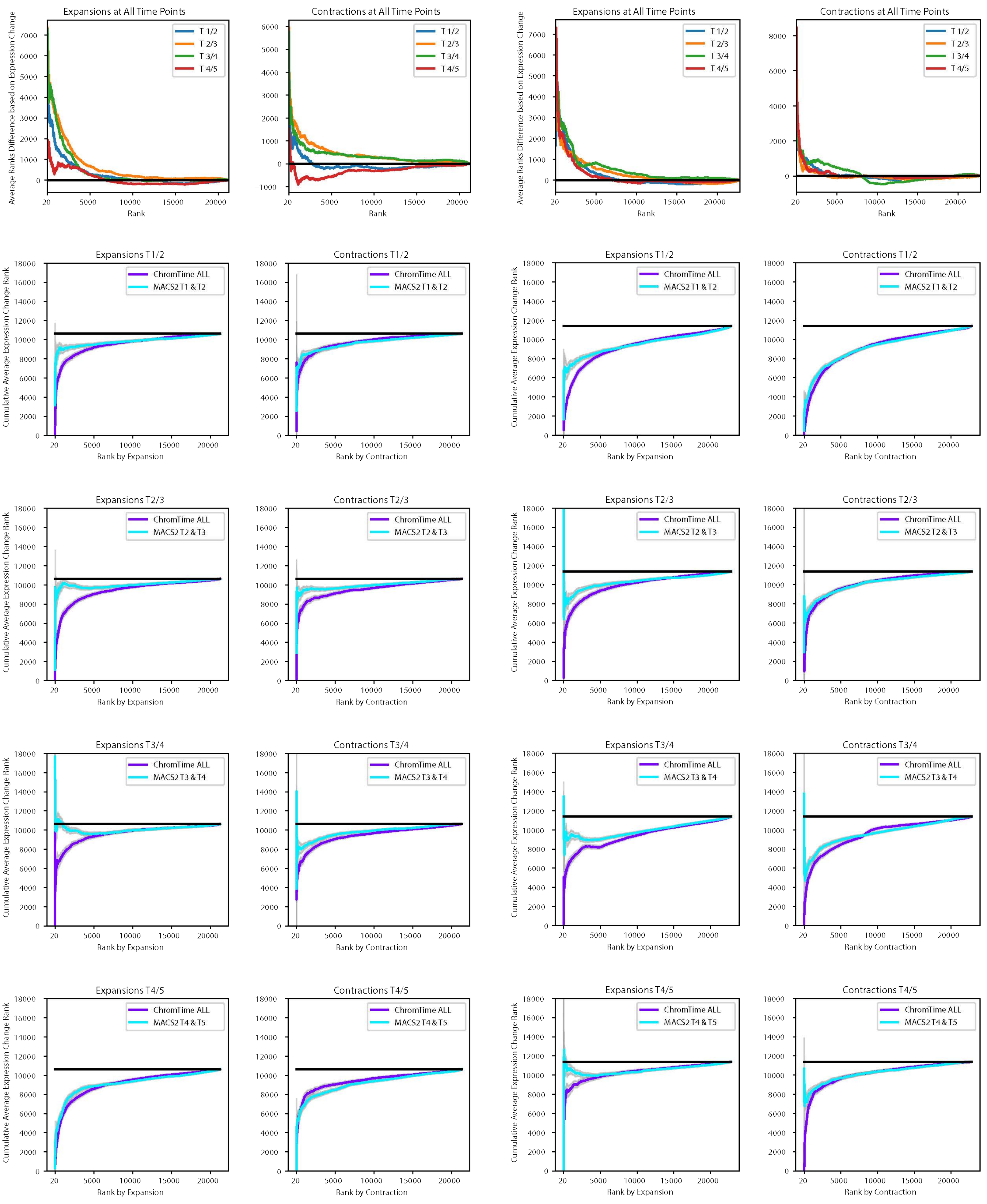

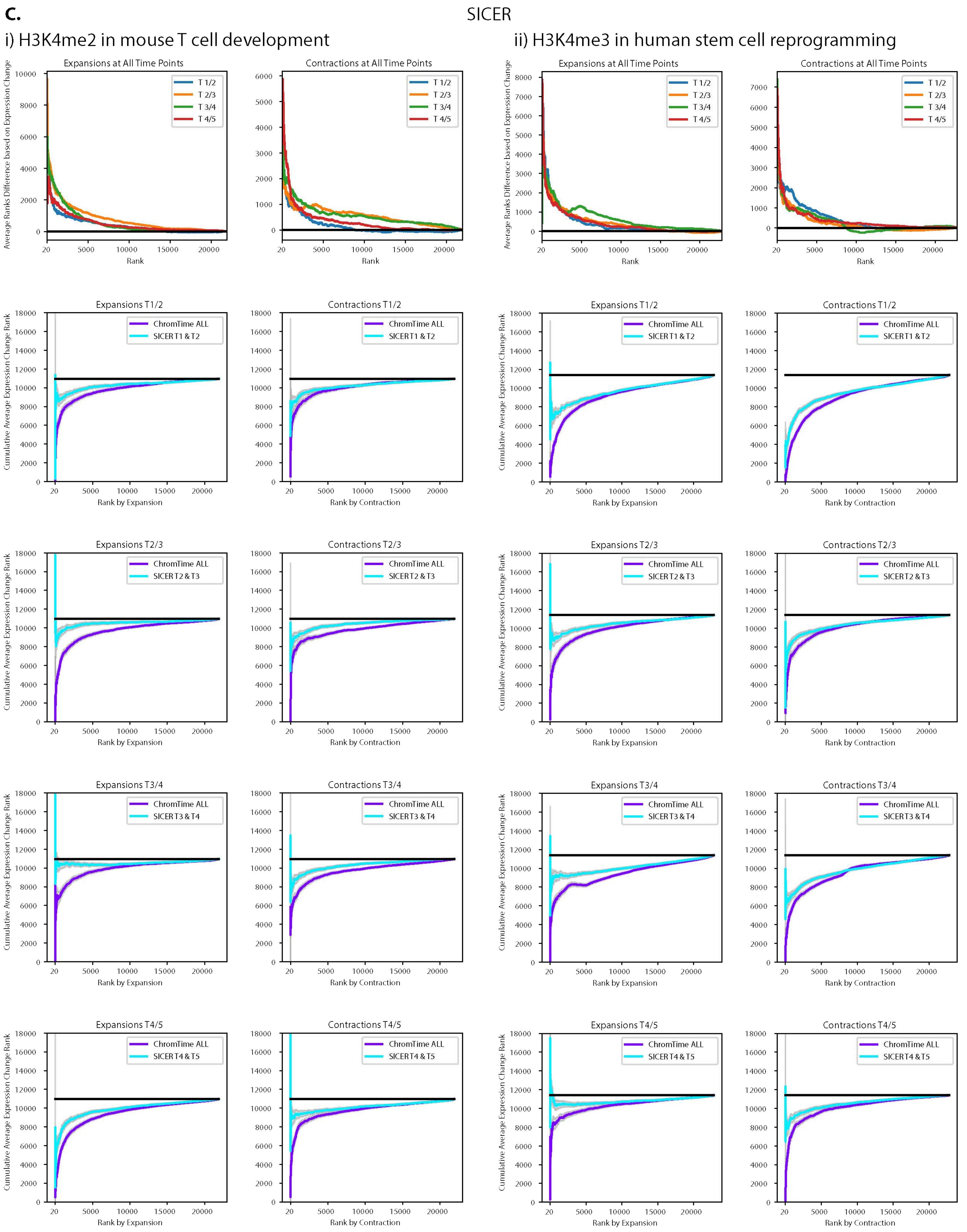

c.
SICER
i) H3K4me2 in mouse T cell development
ii) H3K4me3 in human stem cell reprogramming
Expansions at All Time Points
Contractions at All Time Points
Average Ranks Difference based on Expression Change
Rank
T 1/2
T 2/3
T 3/4
T 4/5
Expansions T1/2
Contractions T1/2
Expansions T2/3
Contractions T2/3
Expansions T3/4
Contractions T3/4
Expansions T4/5
Contractions T4/5
Cumulative Average Expression Change Rank
Rank by Expansion
Rank by Contraction
ChromTime ALL
SICER T1 & T2
SICER T2 & T3
SICER T3 & T4
SICER T4 & T5

Fig S5: Predicted spatial dynamics by ChromTime associate better with gene expression changes compared to boundary position changes of peaks called from single time points in isolation. This figure extends results presented in **Fig 4**. **(A) (i)** For H3K4me2 in mouse T cell development[1] ChromTime was applied once with data from all time points (ChromTime ALL), and once with single time points in isolation (ChromTime SINGLE; see Additional file 2: **Supplementary Methods**). Time points 1, 2 and 3 correspond to T cell precursors, whereas 4 and 5 to purified thymocytes. Peaks called by both procedures overlapping annotated TSSs were analyzed for their relationship with gene expression changes. **(Left)** Plots show comparisons of agreement with expression for expansions when applying ChromTime ALL and ChromTime SINGLE for the change between each pair of time points. Peak boundaries were sorted in decreasing order of their Expand posterior probabilities from ChromTime ALL at each pair of consecutive time points and compared to sorting them in decreasing order of the difference of their positions in ChromTime SINGLE peaks with positive differences indicating peaks expanding with time. Each boundary was also ranked by the average gene expression difference of genes whose TSSs overlap its block in decreasing order with positive expression differences in boundary positions indicating gain with time. The cumulative average boundary rank of expression change was calculated for the boundary change ranking for ChromTime ALL and ChromTime SINGLE (X-axis). The plots in the top row shows the differences between ChromTime ALL and ChromTime SINGLE values shown in the individual plots below for each pair of time points, also shown in **Fig 4Aii**. Positive values correspond to boundary ranks for which ChromTime ALL posteriors better associate with gene expression changes than boundary movements of ChromTime SINGLE peaks. Black lines show expected difference of zero between random rankings. The plots in the subsequent rows show the individual lines used to compute the differences between ChromTime ALL and ChromTime SINGLE rankings shown in the top row and in **Fig 4Aii** for each pair of time points. In particular, cumulative average boundary rank of expression change (Y-axis) is shown for the boundary change ranking for ChromTime ALL and ChromTime SINGLE (X-axis). Low Y-values indicate stronger association with expression changes. Black line shows expected average expression change rank. Shaded regions indicate 95% confidence intervals. **(Right)** Analogous to left plots for Contract posterior probabilities for ChromTime ALL, increasing order of the difference of boundary change positions for ChromTime SINGLE, and increasing order of expression changes. (**ii**) As in (**i**) for H3K4me3 in human stem cell reprogramming[2]. Time points correspond to human inducible and immortalized fibroblasts-like (hiF-T) cells, hiF-T at 5, 10 and 20 days after induction, and human induced pluripotent stem cells (hIPSC). (**B**) as in (**A**) when ChromTime ALL posteriors are compared to boundary movements of MACS2[3] peaks called at single time points in isolation. (**C**) as in (**A-B**) when ChromTime ALL posteriors are compared to boundary movements of SICER peaks called at single time points in isolation.

Fig S6

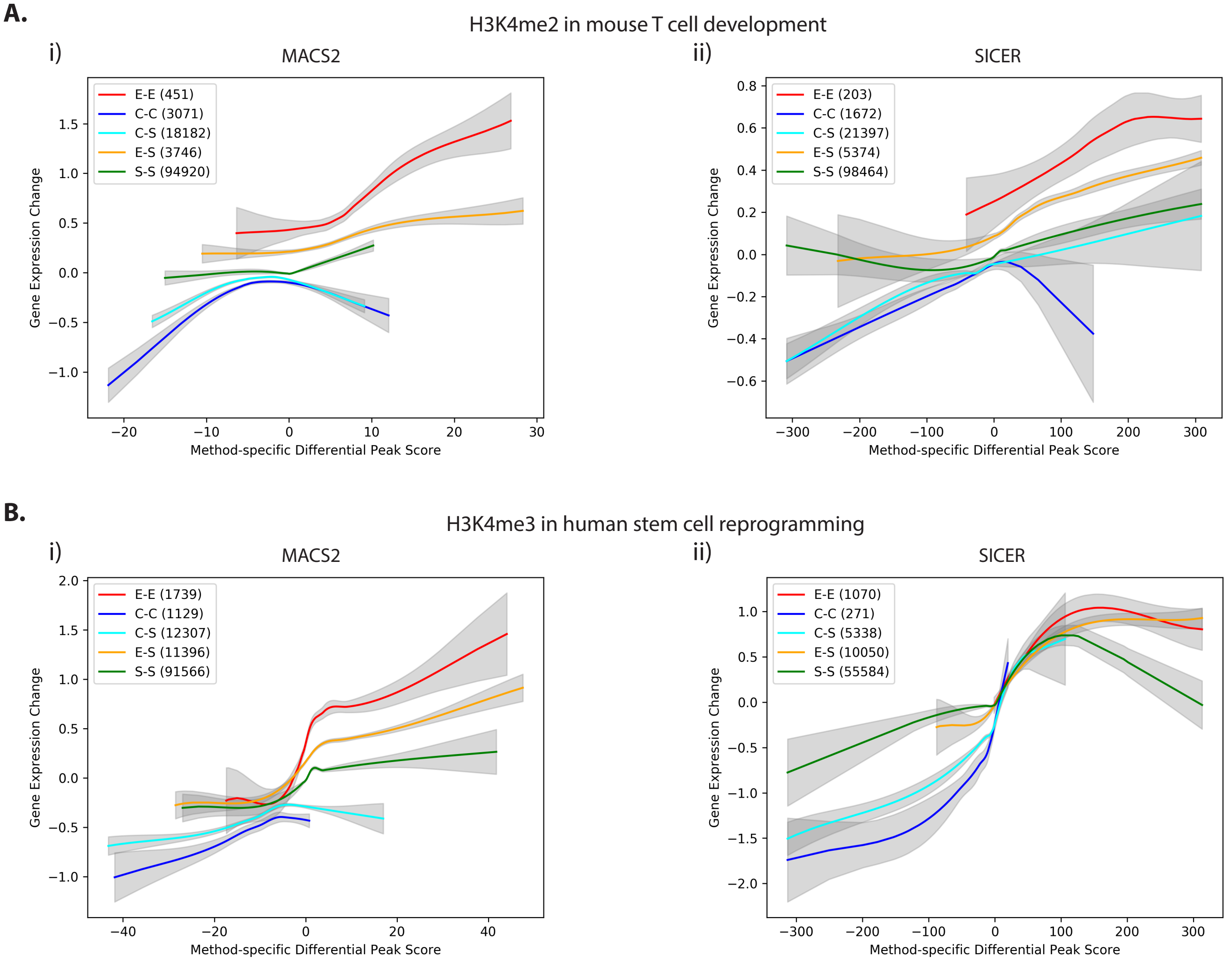

Fig S6: Spatial dynamics can contain additional information about gene expression changes beyond differential peak calls and signal density changes. Similar to **Fig 5**, gene expression change is plotted as function of method-specific differential peak scores after loess smoothing from two differential peak calling methods, MACS2[3] (left) and SICER[6] (right) for each predicted ChromTime dynamic (Additional file 2: **Supplementary Methods**) for **(A)** H3K4me2 dynamics in T cell development in mouse[1]; and **(B)** in H3K4me3 dynamics in stem cell reprogramming in human[2]. Peaks of each type of dynamics were pooled from all time points in each dataset for this analysis. Peaks with asymmetric dynamics E/S and S/E were pooled together in the "E-S" group. Similarly C/S and S/C peaks were pooled in the "C-S" group. In both systems, for a range of differential scores peaks with the same differential score associated with different gene expression changes depending on the predicted spatial dynamic. Shaded regions represent 95% confidence intervals. The number of overlaps between ChromTime peaks and MACS2 or SICER differential peaks for each group is given in parenthesis.

Fig S7

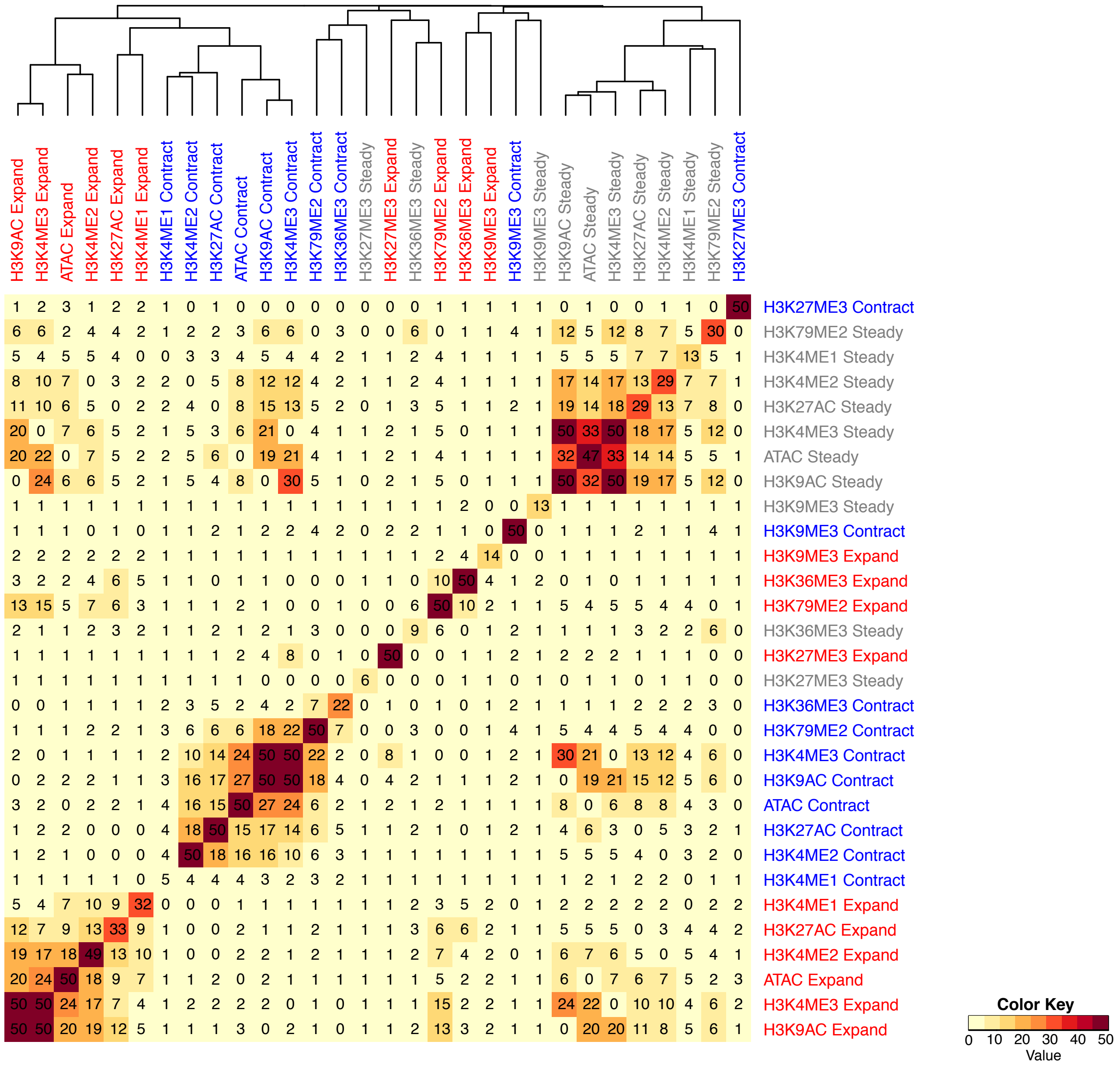

Fig S7: Spatial dynamics of multiple different chromatin marks co-localize within a time course. As in **Fig 6**, hierarchical clustering with optimal leaf ordering[7] of the geometric average of fold enrichments taken across all time points of the overlap of every pair of predicted spatial dynamics for histone modifications and ATAC-seq predicted ChromTime peaks in a mouse stem cell reprogramming time course with four time points[4]. Time points correspond to mouse embryonic fibroblasts, 48 hours after induction of reprogramming, late intermediate pre-induced pluripotent stem cells and mouse embryonic stem cells. At each pair of time points, “Expand” and “Contract” dynamics are defined as all peaks that are predicted as either unidirectional or bidirectional expansions and contractions, respectively, whereas “Steady” dynamics are defined as all peaks that have predicted steady boundaries at both sides. Peaks with “Expand” dynamic on one side and “Contract” dynamic on the other were excluded from this analysis. Predicted expansions, contractions and steady peaks of ATAC-seq, H3K4me2, H3K4me3, H3K27ac, H3K9ac and to a lesser extent of H3K4me1

and H3K79me2 tend to cluster together within each of the three classes, whereas predicted spatial dynamics of H3K27me3, H3K9me3 and H3K36me3 peaks tend to occupy different locations. All enrichments were capped at 50 before clustering.

Fig S8

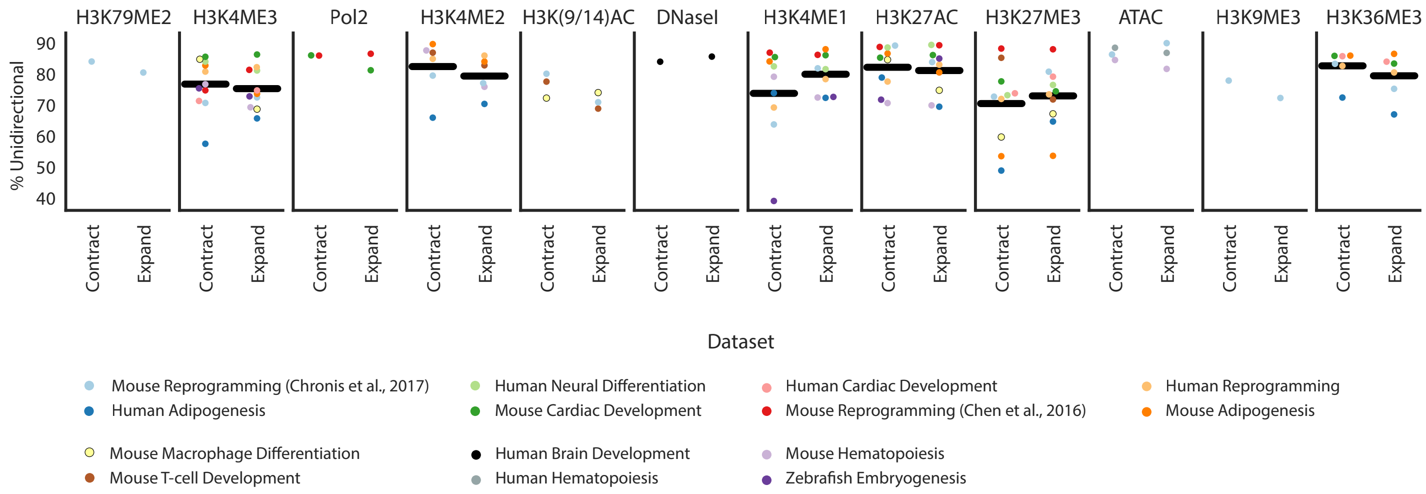

Fig S8: Average percentages of unidirectional expansions and contractions per pair of consecutive time points for each dataset. For each chromatin mark within each time course, the average percentage of unidirectional expansions and contractions out of all expansions and contractions, respectively, per pair of consecutive time points are shown. For marks represented by six or more datasets, the average across all datasets is plotted as a black line.

REFERENCES

1. Zhang JA, Mortazavi A, Williams BA, Wold BJ, Rothenberg E V. Dynamic transformations of genome-wide epigenetic marking and transcriptional control establish T cell identity. Cell. 2012;149:467–82. doi:10.1016/j.cell.2012.01.056.

2. Cacchiarelli D, Trapnell C, Ziller MJ, Soumillon M, Cesana M, Karnik R, et al. Integrative Analyses of Human Reprogramming Reveal Dynamic Nature of Induced Pluripotency. Cell. 2015;162:412–24. doi:10.1016/j.cell.2015.06.016.

3. Zhang Y, Liu T, Meyer CA, Eeckhoute J, Johnson DS, Bernstein BE, et al. Model-based analysis of ChIP-Seq (MACS). Genome Biol. 2008;9:R137. doi:10.1186/gb-2008-9-9-r137.

4. Chronis C, Fiziev P, Papp B, Butz S, Bonora G, Sabri S, et al. Cooperative Binding of Transcription Factors Orchestrates Reprogramming. Cell. 2017;168:442–459.e20. doi:10.1016/j.cell.2016.12.016.

5. Roadmap Epigenomics Consortium, Kundaje A, Meuleman W, Ernst J, Bilenky M, Yen A, et al. Integrative analysis of 111 reference human epigenomes. Nature. 2015;518:317–30. doi:10.1038/nature14248.

6. Xu S, Grullon S, Ge K, Peng W. Spatial clustering for identification of ChIP-enriched regions (SICER) to map regions of histone methylation patterns in embryonic stem cells. Methods Mol Biol. 2014;1150:97–111. doi:10.1007/978-1-4939-0512-6_5.

7. Bar-Joseph Z, Gifford DK, Jaakkola TS. Fast optimal leaf ordering for hierarchical clustering. Bioinformatics. 2001;17 SUPPL. 1:S22-9. doi:10.1093/bioinformatics/17.suppl_1.S22.
