# Supplementary material for: ChromTime: modeling spatio-temporal dynamics of chromatin marks
Source: Genome Biol. 2018 Aug 10;19:109. doi: 10.1186/s13059-018-1485-2 (PMC6085762; doi:10.1186/s13059-018-1485-2)
Supplement: Supplementary file 2 — Further description of methods and analyses in this study. (PDF 711 kb) [file 13059_2018_1485_MOESM2_ESM.pdf]

SUPPLEMENTARY METHODS

ChromTime model optimization

Derivation of objective function

As stated in the **Methods**, the total set of parameters of the model consists of:

1) Prior probabilities of each dynamic *d* at each time point *t*: $\pi_{t,d}$.
2) Parameters of the negative binomial distributions that model the PEAK and the BACKGROUND components at each time point: α_t, β_t, γ_t and δ_t.
3) Parameters of the negative binomial distributions that model the boundary movements in EXPAND and CONTRACT dynamics at each time point: $\mu_{\text{EXPAND,t}}, \delta_{\text{EXPAND,t}}$ and $\mu_{\text{CONTRACT,t}}, \delta_{\text{CONTRACT,t}}$, respectively.

The optimal values of the model parameters are attempted to be estimated by Expectation Maximization (EM). In particular, ChromTime attempts to optimize the expectation of the complete log-likelihood function conditioned on the covariates of all blocks:

$$Q(\theta|\boldsymbol{X}=\boldsymbol{x},\tilde{\theta}) = E_{\boldsymbol{H}|\boldsymbol{O},\boldsymbol{Z},\boldsymbol{X};\tilde{\theta}}\left[\sum_{i=1}^{M}\log P\big(\boldsymbol{O}_i=\boldsymbol{o}_i,\boldsymbol{H}_i,\boldsymbol{Z}_i=\boldsymbol{1}\big|\boldsymbol{X}_i=\boldsymbol{x}_i;\tilde{\theta}\big)\right]$$

$$= \sum_{i=1}^{M} E_{\boldsymbol{H}_i|\boldsymbol{O}_i,\boldsymbol{Z}_i,\boldsymbol{X}_i;\tilde{\theta}}\left[\log P\big(\boldsymbol{O}_i=\boldsymbol{o}_i,\boldsymbol{H}_i,\boldsymbol{Z}_i=\boldsymbol{1}\big|\boldsymbol{X}_i=\boldsymbol{x}_i;\tilde{\theta}\big)\right]$$

where θ is the set of all model parameters that are attempted to be optimized, $\tilde{\theta}$ is the set of the EM algorithm's current values for θ, the index i iterates over all M blocks in the dataset. $\boldsymbol{H}_i$ is the set of all latent variables in the model (Additional file 1: **Fig S1C**) which includes all $B_{i,L,t}, D_{i,L,t}, B_{i,R,t}, D_{i,R,t}$ and $V_{i,t,p}$ variables. $\boldsymbol{Z}_i$ is the set of all $Z_{i,t}$ variables in block i which enforce that the peak boundaries are within the range of the block and that the left end boundaries, $B_{i,L,t}$ are placed before right end boundaries, $B_{i,R,t}$. $\boldsymbol{O}_i$

and $\boldsymbol{X}_i$ are the sets of the variables that model the observed read counts and their covariates, respectively, at all bins and time points in block i. $\boldsymbol{o}_i$ and $\boldsymbol{x}_i$ are the corresponding values of the observed read counts and their covariates, respectively. The above expectation is taken with respect to all latent variables, conditioned on the values of all observed variables and the current values of $\tilde{\theta}$.

Let $\mathbf{W}_i$ and $\mathbf{w}_i$ denote the set of all observed variables in the model for block i, which include $\boldsymbol{O}_i$, $\boldsymbol{X}_i$ and $\boldsymbol{Z}_i$, and their values, respectively. Then,

$$E_{\boldsymbol{H}_i|\boldsymbol{O}_i,\boldsymbol{Z}_i,\boldsymbol{X}_i;\tilde{\theta}}\left[\log P\left(\boldsymbol{O}_i = \boldsymbol{o}_i, \boldsymbol{H}_i, \boldsymbol{Z}_i = \mathbf{1} \middle| \boldsymbol{X}_i = \boldsymbol{x}_i; \tilde{\theta}\right)\right]$$

$$= E_{\boldsymbol{H}_i|\boldsymbol{W}_i;\tilde{\theta}}\left[\log P\left(\boldsymbol{O}_i = \boldsymbol{o}_i, \boldsymbol{H}_i, \boldsymbol{Z}_i = \mathbf{1} \middle| \boldsymbol{X}_i = \boldsymbol{x}_i; \tilde{\theta}\right)\right]$$

$$= \sum_{l_1=1}^{N_i+1} \sum_{r_1=l_1-1}^{N_i} P\left(B_{i,L,1} = l_1, B_{i,R,1} = r_1 | \mathbf{W}_i = \mathbf{w}_i; \tilde{\theta}\right)$$

$$\times \left(\sum_{p=1}^{l_1-1} \log P\left(O_{i,1,p} = o_{i,1,p} \middle| V_{i,1,p} = \text{BACKGROUND}, X_{i,1,p} = x_{i,1,p}\right) \right.$$

$$+ \sum_{p=l_1}^{r_1} \log P\left(O_{i,1,p} = o_{i,1,p} \middle| V_{i,1,p} = \text{PEAK}, X_{i,1,p} = x_{i,1,p}\right)$$

$$+ \sum_{p=r_1+1}^{N_i} \log P\left(O_{i,1,p} = o_{i,1,p} \middle| V_{i,1,p} = \text{BACKGROUND}, X_{i,1,p} = x_{i,1,p}\right) + \log P\left(B_{i,L,1} = l_1\right)$$

$$\left. + \log P\left(B_{i,R,1} = r_1\right) \right)$$

$$+ \sum_{t=2}^{T} \sum_{d_L \in \mathbb{D}} \sum_{d_R \in \mathbb{D}} \sum_{l_t=1}^{N_i+1} \sum_{r_t=l_t-1}^{N_i} \sum_{l_{t-1}=1}^{N_i+1} \sum_{r_{t-1}=l_{t-1}-1}^{N_i}$$

$$\mathrm{P}\left(B_{i,L,t} = l_t, B_{i,L,t-1} = l_{t-1}, D_{i,L,t-1} = d_L, B_{i,R,t} = r_t, B_{i,R,t-1} = r_{t-1}, D_{i,R,t-1} = d_R \middle| \mathbf{W}_i = \mathbf{w}_i; \tilde{\theta}\right)$$

$$\times\Bigg(\Bigg(\log P\big(B_{i,L,t} = l_t | B_{i,L,t-1} = l_{t-1}, D_{i,L,t-1} = d_L\big) + \log P(B_{i,R,t} = r_t | B_{i,R,t-1} = r_{t-1}, D_{i,R,t-1} = d_R)$$

$$+ \log P\big(D_{i,L,t-1} = d_L\big) + \log P\big(D_{i,R,t-1} = d_R\big)$$

$$+ \sum_{p=1}^{l_t-1} \log P\big(O_{i,t,p} = o_{i,t,p} \big| V_{i,t,p} = \mathrm{BACKGROUND}, X_{i,t,p} = x_{i,t,p}\big)$$

$$+ \sum_{p=l_t}^{r_t} \log P\big(O_{i,t,p} = o_{i,t,p} \big| V_{i,t,p} = \mathrm{PEAK}, X_{i,t,p} = x_{i,t,p}\big)$$

$$+ \sum_{p=r_t+1}^{N_i} \log P\big(O_{i,t,p} = o_{i,t,p} \big| V_{i,t,p} = \mathrm{BACKGROUND}, X_{i,t,p} = x_{i,t,p}\big)\Bigg)\Bigg)$$

where T denotes the number of time points in the time course, $o_{i,t,p}$ and $x_{i,t,p}$ denote the number of observed foreground reads and covariates, respectively, in block i at time point t at position p, N_i denotes the number of bins in block i and $\mathbb{D}$ denotes the set of all dynamics ($\mathbb{D} = \{\mathrm{STEADY}, \mathrm{EXPAND}, \mathrm{CONTRACT}\}$).

The expectation of the complete log likelihood, $Q\big(\theta | \boldsymbol{X} = \boldsymbol{x}, \tilde{\theta}\big)$, simplifies substantially, if we substitute in the above equation each of the following terms:

$$\sum_{l_1=1}^{N_i+1} \sum_{r_1=l_1-1}^{N_i} P\big(B_{i,L,1} = l_1, B_{i,R,1} = r_1 | \mathbf{W}_i = \mathbf{w}_i; \tilde{\theta}\big)$$

$$\times\Bigg(\sum_{p=1}^{l_1-1} \log P\big(O_{i,1,p} = o_{i,1,p} \big| V_{i,1,p} = \mathrm{BACKGROUND}, X_{i,1,p} = x_{i,1,p}\big)$$

$$+ \sum_{p=l_1}^{r_1} \log P\big(O_{i,1,p} = o_{i,1,p} \big| V_{i,1,p} = \mathrm{PEAK}, X_{i,1,p} = x_{i,1,p}\big)$$

$$+ \sum_{p=r_1+1}^{N_i} \log P\big(O_{i,1,p} = o_{i,1,p} \big| V_{i,1,p} = \mathrm{BACKGROUND}, X_{i,1,p} = x_{i,1,p}\big)\Bigg)$$

$$= \sum_{p=1}^{N_i} \log P\big(O_{i,1,p} = o_{i,1,p} \big| V_{i,1,p} = \mathrm{PEAK}, X_{i,1,p} = x_{i,1,p}\big) \sum_{l_1=1}^{p} \sum_{r_1=p}^{N_i} P\big(B_{i,L,1} = l_1, B_{i,R,1} = r_1 | \mathbf{W}_i = \mathbf{w}_i; \tilde{\theta}\big)$$

$$+\sum_{p=1}^{N_i} \log P\big(O_{i,1,p} = o_{i,1,p}\big|V_{i,1,p} = \text{BACKGROUND}, X_{i,1,p} = x_{i,1,p}\big)$$

$$\times\left(\sum_{l_1=1}^{p-1}\sum_{r_1=l_1-1}^{p-1} P\big(B_{i,L,1} = l_1, B_{i,R,1} = r_1 | \mathbf{W}_i = \mathbf{w}_i; \tilde{\theta}\big) + \sum_{l_1=p+1}^{N_i+1}\sum_{r_1=l_1-1}^{N_i} P\big(B_{i,L,1} = l_1, B_{i,R,1} = r_1 | \mathbf{W}_i = \mathbf{w}_i; \tilde{\theta}\big)\right)$$

$$=\sum_{p=1}^{N_i} \Big(P\big(V_{i,1,p} = \text{PEAK}\big|\mathbf{W}_i = \mathbf{w}_i; \tilde{\theta}\big) \log P\big(O_{i,1,p} = o_{i,1,p}\big|V_{i,1,p} = \text{PEAK}, X_{i,1,p} = x_{i,1,p}\big)$$

$$+\Big(1 - P\big(V_{i,1,p} = \text{PEAK}\big|\mathbf{W}_i = \mathbf{w}_i; \tilde{\theta}\big)\Big) \log P\big(O_{i,1,p} = o_{i,1,p}\big|V_{i,1,p} = \text{BACKGROUND}, X_{i,1,p} = x_{i,1,p}\big)\Big)$$

$$=\sum_{p=1}^{N_i} \Big(P\big(V_{i,1,p} = \text{PEAK}\big|\mathbf{W}_i = \mathbf{w}_i; \tilde{\theta}\big) \log\Big(\text{NB}\big(o_{i,1,p}; \mu_{\text{PEAK},1} = \exp[\alpha_1 + \gamma_1 \log \lambda_{i,1,p}], \delta_1\big)\Big)$$

$$+ P\big(V_{i,1,p} = \text{BACKGROUND}\big|\mathbf{W}_i = \mathbf{w}_i; \tilde{\theta}\big) \log\Big(\text{NB}\big(o_{i,1,p}; \mu_{\text{BACKGROUND},1} = \exp[\beta_1 + \gamma_1 \log \lambda_{i,1,p}], \delta_1\big)\Big)\Big)$$

and

$$\sum_{t=2}^{T}\sum_{d_L\in\mathbb{D}}\sum_{d_R\in\mathbb{D}}\sum_{l_t=1}^{N_i+1}\sum_{r_t=l_t-1}^{N_i}\sum_{l_{t-1}=1}^{N_i+1}\sum_{r_{t-1}=l_{t-1}-1}^{N_i}$$

$$\text{P}\big(B_{i,L,t} = l_t, B_{i,L,t-1} = l_{t-1}, D_{i,L,t-1} = d_L, B_{i,R,t} = r_t, B_{i,R,t-1} = r_{t-1}, D_{i,R,t-1} = d_R\big|\mathbf{W}_i = \mathbf{w}_i; \tilde{\theta}\big)$$

$$\times\ \big(\log P\big(B_{i,L,t} = l_t | B_{i,L,t-1} = l_{t-1}, D_{i,L,t-1} = d_L\big) + \log P(B_{i,R,t} = r_t | B_{i,R,t-1} = r_{t-1}, D_{i,R,t-1} = d_R)\big)$$

$$=\sum_{t=2}^{T}\sum_{d_L\in\mathbb{D}}\sum_{l_{t-1}=1}^{N_i+1}\sum_{l_t=1}^{N_i+1} \text{P}\big(B_{i,L,t} = l_t, B_{i,L,t-1} = l_{t-1}, D_{i,L,t-1} = d_L\big|\mathbf{W}_i = \mathbf{w}_i; \tilde{\theta}\big)$$

$$\times \log P\big(B_{i,L,t} = l_t | B_{i,L,t-1} = l_{t-1}, D_{i,L,t-1} = d_L\big)$$

$$+\sum_{t=2}^{T}\sum_{d_R\in\mathbb{D}}\sum_{r_{t-1}=0}^{N_i}\sum_{r_t=0}^{N_i} \text{P}\big(B_{i,R,t} = r_t, B_{i,R,t-1} = r_{t-1}, D_{i,R,t-1} = d_R\big|\mathbf{W}_i = \mathbf{w}_i; \tilde{\theta}\big)$$

$$\times \log P\big(B_{i,R,t} = r_t | B_{i,R,t-1} = r_{t-1}, D_{i,R,t-1} = d_R\big)$$

$$=\sum_{t=1}^{T-1}\sum_{s\in\{L,R\}}\sum_{d\in\left\{\substack{\text{EXPAND,}\\ \text{CONTRACT}}\right\}}\sum_{j=1}^{N_i} P\big(J_{i,s,t} = (-1)^{g(d)} j, D_{i,s,t} = d\big|\mathbf{W}_i = \mathbf{w}_i; \tilde{\theta}\big)$$

$$\times \log\Big(P\big(J_{i,s,t} = (-1)^{g(d)} j | D_{i,s,t} = d\big)\Big)$$

$$= \sum_{t=1}^{T-1} \sum_{s \in \{L,R\}} \sum_{d \in \left\{ \substack{\text{EXPAND,} \\ \text{CONTRACT}} \right\}} \sum_{j=1}^{N_i} P\left(J_{i,s,t} = (-1)^{g(d)} j, D_{i,s,t} = d \middle| \mathbf{W}_i = \mathbf{w}_i; \tilde{\theta}\right) \log\left(\text{NB}(j-1; \mu_d, \delta_d)\right)$$

where

$$g(d) = \begin{cases} 1 & \text{if } d = \text{CONTRACT} \\ 0 & \text{otherwise} \end{cases}$$

In the above we used the simplification that summing over all possible ways to place the peak boundaries on the left and on the right side at two consecutive time points is equivalent to summing over all possible distances between two left boundaries and all possible distances between two right boundaries, *j*.

Also, we can simplify:

$$\sum_{t=2}^{T} \sum_{d_L \in \mathbb{D}} \sum_{d_R \in \mathbb{D}} \sum_{l_t=1}^{N_i+1} \sum_{r_t=l_t-1}^{N_i} \sum_{l_{t-1}=1}^{N_i+1} \sum_{r_{t-1}=l_{t-1}-1}^{N_i}$$

$$\text{P}\left(B_{i,L,t} = l_t, B_{i,L,t-1} = l_{t-1}, D_{i,L,t-1} = d_L, B_{i,R,t} = r_t, B_{i,R,t-1} = r_{t-1}, D_{i,R,t-1} = d_R \middle| \mathbf{W}_i = \mathbf{w}_i; \tilde{\theta}\right)$$

$$\times \left(\log P\left(D_{i,L,t-1} = d_L\right) + \log P\left(D_{i,R,t-1} = d_R\right)\right)$$

$$= \sum_{t=1}^{T-1} \sum_{s \in \{L,R\}} \sum_{d \in \left\{ \substack{\text{STEADY,} \\ \text{EXPAND,} \\ \text{CONTRACT}} \right\}} P\left(D_{i,s,t} = d \middle| \mathbf{W}_i = \mathbf{w}_i; \tilde{\theta}\right) \log\left(P\left(D_{i,s,t} = d\right)\right)$$

$$= \sum_{t=1}^{T-1} \sum_{s \in \{L,R\}} \sum_{d \in \left\{ \substack{\text{STEADY,} \\ \text{EXPAND,} \\ \text{CONTRACT}} \right\}} P\left(D_{i,s,t} = d \middle| \mathbf{W}_i = \mathbf{w}_i; \tilde{\theta}\right) \log\left(\pi_{t,d}\right)$$

and

$$\sum_{t=2}^{T} \sum_{d_L \in \mathbb{D}} \sum_{d_R \in \mathbb{D}} \sum_{l_t=1}^{N_i+1} \sum_{r_t=l_t-1}^{N_i} \sum_{l_{t-1}=1}^{N_i+1} \sum_{r_{t-1}=l_{t-1}-1}^{N_i}$$

$$\text{P}\left(B_{i,L,t} = l_t, B_{i,L,t-1} = l_{t-1}, D_{i,L,t-1} = d_L, B_{i,R,t} = r_t, B_{i,R,t-1} = r_{t-1}, D_{i,R,t-1} = d_R \middle| \mathbf{W}_i = \mathbf{w}_i; \tilde{\theta}\right)$$

$$\times\left(\sum_{p=1}^{l_t-1}\log P\big(O_{i,t,p}=o_{i,t,p}\big|V_{i,t,p}=\text{BACKGROUND},X_{i,t,p}=x_{i,t,p}\big)\right.$$

$$+\sum_{p=l_t}^{r_t}\log P\big(O_{i,t,p}=o_{i,t,p}\big|V_{i,t,p}=\text{PEAK},X_{i,t,p}=x_{i,t,p}\big)$$

$$\left.+\sum_{p=r_t+1}^{N_i}\log P\big(O_{i,t,p}=o_{i,t,p}\big|V_{i,t,p}=\text{BACKGROUND},X_{i,t,p}=x_{i,t,p}\big)\right)$$

$$=\sum_{t=2}^{T}\sum_{p=1}^{N_i}\Big(P\big(V_{i,t,p}=\text{PEAK}\big|\mathbf{W}_i=\mathbf{w}_i;\tilde{\theta}\big)\log P\big(O_{i,t,p}=o_{i,t,p}\big|V_{i,t,p}=\text{PEAK},X_{i,t,p}=x_{i,t,p}\big)$$

$$+P\big(V_{i,t,p}=\text{BACKGROUND}\big|\mathbf{W}_i=\mathbf{w}_i;\tilde{\theta}\big)\log P\big(O_{i,t,p}=o_{i,t,p}\big|V_{i,t,p}=\text{BACKGROUND},X_{i,t,p}=x_{i,t,p}\big)\Big)$$

$$=\sum_{t=1}^{T}\sum_{p=1}^{N_i}\Big(P\big(V_{i,t,p}=\text{PEAK}\big|\mathbf{W}_i=\mathbf{w}_i;\tilde{\theta}\big)\log\Big(\text{NB}\big(o_{i,t,p};\mu_{\text{PEAK},t}=\exp\big[\alpha_t+\gamma_t\log\lambda_{i,t,p}\big],\delta_t\big)\Big)$$

$$+P\big(V_{i,t,p}=\text{BACKGROUND}\big|\mathbf{W}_i=\mathbf{w}_i;\tilde{\theta}\big)\log\Big(\text{NB}\big(o_{i,t,p};\mu_{\text{BACKGROUND},t}=\exp\big[\beta_t+\gamma_t\log\lambda_{i,t,p}\big],\delta_t\big)\Big)\Big)$$

With these substitutions, we can rewrite the expectation of the conditional complete log likelihood of the data, $Q(\theta|\boldsymbol{X}=\boldsymbol{x},\tilde{\theta})$, as

$$\sum_{i=1}^{M}\sum_{l_1=1}^{N_i+1}\sum_{r_1=l_1-1}^{N_i}P\big(B_{i,L,1}=l_1,B_{i,R,1}=r_1|\mathbf{W}_i=\mathbf{w}_i;\tilde{\theta}\big)\big(\log P\big(B_{i,L,1}=l_1\big)+\log P\big(B_{i,R,1}=r_1\big)\big)$$

$$+\sum_{i=1}^{M}\sum_{t=1}^{T}\sum_{p=1}^{N_i}\Big(P\big(V_{i,t,p}=\text{PEAK}\big|\mathbf{W}_i=\mathbf{w}_i;\tilde{\theta}\big)\log\Big(\text{NB}\big(o_{i,t,p};\mu_{\text{PEAK},t}=\exp\big[\alpha_t+\gamma_t\log\lambda_{i,t,p}\big],\delta_t\big)\Big)$$

$$+\ P\big(V_{i,t,p}=\text{BACKGROUND}\big|\mathbf{W}_i=\mathbf{w}_i;\tilde{\theta}\big)\log\Big(\text{NB}\big(o_{i,t,p};\mu_{\text{BACKGROUND},t}=\exp\big[\beta_t+\gamma_t\log\lambda_{i,t,p}\big],\delta_t\big)\Big)\Big)$$

$$+\sum_{i=1}^{M}\sum_{t=1}^{T-1}\sum_{s\in\{L,R\}}\sum_{d\in\left\{\substack{\text{EXPAND,}\\ \text{CONTRACT}}\right\}}\sum_{j=1}^{N_i}P\big(J_{i,s,t}=(-1)^{g(d)}j,D_{i,s,t}=d\big|\mathbf{W}_i=\mathbf{w}_i;\tilde{\theta}\big)\times\log\big(\text{NB}(j-1;\mu_d,\delta_d)\big)$$

$$+\sum_{i=1}^{M}\sum_{t=1}^{T-1}\sum_{s\in\{L,R\}}\sum_{d\in\left\{\substack{\text{STEADY,}\\ \text{EXPAND,}\\ \text{CONTRACT}}\right\}}P\big(D_{i,s,t}=d\big|\mathbf{W}_i=\mathbf{w}_i;\tilde{\theta}\big)\log\big(\pi_{t,d}\big)$$

Parameter initialization

The values of the model parameters before the first EM iteration are initialized as follows:

1) All dynamics priors, $\pi_{t,d}$, are set uniformly to $\frac{1}{3}$;
2) All parameters for the distributions modelling the PEAK and BACKGROUND components, α_t, β_t, γ_t and δ_t, are set to 1;
3) All dispersion and mean parameters for the distributions modelling the boundary movements in EXPAND and CONTRACT dynamics, $\mu_{\text{EXPAND,t}}$, $\delta_{\text{EXPAND,t}}$, $\mu_{\text{CONTRACT,t}}$, and $\delta_{\text{CONTRACT,t}}$, are set to 1.

At each time point, ChromTime requires that the position of the left boundary of each peak is placed before the position of the right boundary (i.e. $B_{i,L,t} \leq B_{i,R,t} + 1$), and that $1 \leq B_{i,L,t} \leq N_i + 1$ and $0 \leq B_{i,R,t} \leq N_i$. For each block i, this requirement is implemented by introducing additional $Z_{i,t}$ variables in the model, which are treated as observed, and setting:

$$P(Z_{i,t} = 1 | B_{i,L,t} = l, B_{i,R,t} = r) = \begin{cases} 1 & \text{if } 1 \leq l \leq r + 1 \leq N + 1 \\ 0 & \text{otherwise} \end{cases}$$

This requirement in combination with the uniform priors over the left and the right peak boundary positions at the first time point, $B_{i,L,1}$ and $B_{i,R,1}$, induces non-uniform conditional probabilities $P(V_{i,t,p} = \text{PEAK} | Z_{i,t} = 1)$ for the $V_{i,t,p}$ variables, which model the probability that a bin at position p and time point t is assigned to the peak component. As a result, bins in the middle of the block are more likely to be in the peak component compared to flanking bins on each side. For example in block i of length N_i in a dataset with only one time point, the conditional probability for a bin at position p ($1 \leq p \leq N_i$) to be in a peak after marginalizing out all other latent variables and the observed read counts in the model can be expressed as a function of p:

$P(V_{i,1,p} = \mathrm{PEAK}|Z_{i,1} = 1)$

$$= \sum_{l=1}^{p} \sum_{r=p}^{N_i} P(B_{i,L,1} = l, B_{i,R,1} = r | Z_{i,1} = 1)$$

$$= \sum_{l=1}^{p} \sum_{r=p}^{N_i} \frac{P(Z_{i,1} = 1 | B_{i,L,1} = l, B_{i,R,1} = r) P(B_{i,L,1} = l, B_{i,R,1} = r)}{P(Z_{i,1} = 1)}$$

$$= \sum_{l=1}^{p} \sum_{r=p}^{N_i} \frac{P(Z_{i,1} = 1 | B_{i,L,1} = l, B_{i,R,1} = r) P(B_{i,L,1} = l) P(B_{i,R,1} = r)}{\sum_{l'=1}^{N_i+1} \sum_{r'=0}^{N_i} P(Z_{i,1} = 1 | B_{i,L,1} = l', B_{i,R,1} = r') P(B_{i,L,1} = l') P(B_{i,R,1} = r')}$$

$$= \sum_{l=1}^{p} \sum_{r=p}^{N_i} \frac{1}{\sum_{l'=1}^{N_i+1} \sum_{r'=l'-1}^{N_i} 1} = \frac{2p(N_i - p + 1)}{(N_i + 1)(N_i + 2)}$$

The above equalities follow from $P(B_{i,L,1} = l') = P(B_{i,R,1} = r') = \frac{1}{N_i+1}$ for all $l' \in [1, N_i + 1], r' \in [0, N_i]$ and $P(Z_{i,1} = 1 | B_{i,L,1} = l', B_{i,R,1} = r') = 1$ for $l' \leq r' - 1$ and $P(Z_{i,1} = 1 | B_{i,L,1} = l', B_{i,R,1} = r') = 0$, otherwise. Of note, $P(V_{i,1,p} = \mathrm{PEAK}|Z_{i,1} = 1)$ as a function of p is symmetric with respect to the center of the block. For even N_i, the maximum of $P(V_{i,1,p} = \mathrm{PEAK}|Z_{i,1} = 1)$ is equal to $\frac{N_i}{2(N_i+1)}$ and is obtained for $p = \frac{N_i}{2}$ and for $p = \frac{N_i}{2} + 1$. For odd N_i, the maximum of $P(V_{i,1,p} = \mathrm{PEAK}|Z_{i,1} = 1)$ is equal to $\frac{N_i+1}{2(N_i+2)}$ and is obtained for $p = \left\lceil \frac{N_i}{2} \right\rceil$. In both cases the maximum is less than $\frac{1}{2}$.

We note that the above function of p is not in itself a probability distribution over p, because p is not a random variable in the model and thus the sum over p does not have to be equal to 1. We have empirically confirmed that in a dataset with five time points $P(V_{i,t,p} = \mathrm{PEAK}|Z_{i,t} = 1)$ has a similar relationship to the position index p, as in datasets with one time point. We did this by marginalizing out the observed read counts and all latent variables except $V_{i,t,p}$ from the probability distribution of all variables in the model conditioned on the block's covariates (**Methods**, Additional file 1: **Fig S1Di**). The conditional probabilities $P(V_{i,t,p} = \mathrm{PEAK}|Z_{i,t} = 1)$ play a role during the learning stage of ChromTime, because they direct the model during the initial iterations of the EM to correctly associate the peak component with high signal and the background component with low signal. The assumption that high

signal will more likely be located in the middle of blocks is motivated by the procedure that determines the block boundaries in the first phase of ChromTime, which naturally produces blocks with more significantly enriched bins in the middle compared to their flanking regions (Additional file 1: **Fig S1Dii**). As a result, no further efforts from the initialization or the training procedures are necessary in practice to identify correctly each component.

Expectation step

ChromTime provides an efficient implementation of the expectation step of EM based on a dynamic programming algorithm similar to the Baum-Welch algorithm for hidden Markov models. In brief, at each time point there are $O(N^2)$ ways to place the start and end positions of a peak, resulting in $O(N^4)$ combinations between any pair of consecutive time points. Thus, a standard forward-backward procedure that caches intermediate results can compute all expectations in $O(T*N^4)$ time and $O(T*N^2)$ memory. Since $O(T*N^4)$ time complexity can result in very long running times even for moderate N, ChromTime splits blocks that are longer than a predefined number of bins, MAX_BINS, (30 by default) into two halves (left and right) and estimates all sufficient statistics in each half independently. If block i is longer than MAX_BINS, the split is performed at the position with the highest average signal across all time points in the block, K_i. This splitting procedure corresponds to imposing an additional constraint on the values of the boundary position variables that $B_{i,L,t} \leq K_i$ and $B_{i,R,t} \geq K_i - 1$ at each time point, t, while still having all bins between the left and the right boundaries annotated as peak bins (i.e. $V_{i,t,p} = \text{PEAK}$ for all p such that $B_{i,L,t} \leq p \leq B_{i,R,t}$ and $V_{i,t,p} = \text{BACKGROUND}$ for all other values of p). This heuristic reduces the time complexity to $O(T*N^2)$ and the memory footprint to $O(T*N)$, thus making the whole EM procedure run in feasible time and space. Since the $O(T*N^4)$ algorithm is applied only to blocks shorter than MAX_BINS bins, the total running time of ChromTime in a dataset of M blocks remains at most quadratic in the length of longer peaks in the data, $O(M*T*N^2)$.

For computational efficiency, if there are more than 10,000 blocks, ChromTime randomly selects 10,000 as input for the EM procedure.

Maximization step

The form of the complete log-likelihood implies that each set of model parameters can be optimized independently by solving for the roots of the respective partial derivatives. The dynamics prior probabilities are updated after each EM iteration as:

$$P(D_{i,L,t} = d) = P(D_{i,R,t} = d) = \frac{1}{2M}\sum_{i=1}^{M}\sum_{s\in\{L,R\}} P(D_{i,s,t} = d|\mathbf{W}_i = \mathbf{w}_i; \tilde{\theta})$$

Users have the option to set a minimum prior probability (MIN_PRIOR) for the dynamics at each time point. This parameter can be used to avoid learning priors too close to zero, which in some cases can occur for more punctate marks where the short length of the peaks can cause the prior to become a dominant influence on the class assignment of the spatial dynamics. By default, MIN_PRIOR=0 in narrow and broad modes and MIN_PRIOR=0.05 in punctate mode. Priors whose updates from the above equation are less than MIN_PRIOR are set to MIN_PRIOR, and the priors for the rest of the dynamics at the same pair of time points are rescaled proportionally to reflect this change. If rescaling causes other priors to be set below MIN_PRIOR, then these priors are also set to MIN_PRIOR and rescaling is repeated until all priors are at least equal to MIN_PRIOR.

Optimizing the peak and background signal components

The part of the expectation of the complete log likelihood that pertains to the peak and background signal components is:

$$\sum_{i=1}^{M}\sum_{t=1}^{T}\sum_{p=1}^{N_i} P(V_{i,t,p} = \mathrm{PEAK}|\mathbf{W}_i = \mathbf{w}_i; \tilde{\theta}) \log\left(\mathrm{NB}(o_{i,t,p}; \mu_{\mathrm{PEAK},t} = \exp[\alpha_t + \gamma_t \log \lambda_{i,t,p}], \delta_t)\right) +$$

$$+\sum_{i=1}^{M}\sum_{t=1}^{T}\sum_{p=1}^{N_i} P\left(V_{i,t,p} = \text{BACKGROUND}\middle|\mathbf{W}_i = \mathbf{w}_i;\tilde{\theta}\right)$$

$$\times \log\left(\text{NB}\left(o_{i,t,p};\mu_{\text{BACKGROUND},t} = \exp\left[\beta_t + \gamma_t \log \lambda_{i,t,p}\right],\delta_t\right)\right)$$

These equations are equivalent to the equations for finding the maximum likelihood estimates for the coefficients and the dispersion parameter of one weighted negative binomial regression for each time point and component (peak and background) that aims to predict the observed number of foreground reads, $o_{i,t,p}$ (as a response), from the vector of covariates $x_{i,t,p} = \left[1, \log \lambda_{i,t,p}\right]$. The coefficients in our case are α_t and γ_t (for the peak component) and β_t and γ_t (for the background component). The weights for each regression correspond to the posterior probabilities $P\left(V_{i,t,p} = \text{PEAK}\middle|\mathbf{W}_i = \mathbf{w}_i;\tilde{\theta}\right)$ and $P\left(V_{i,t,p} = \text{BACKGROUND}\middle|\mathbf{W}_i = \mathbf{w}_i;\tilde{\theta}\right)$, respectively. In contrast to standard negative binomial regression, for each time point we have a pair of coupled negative binomial regressions that share the dispersion parameter δ_t and the coefficient γ_t. ChromTime implements a procedure that jointly optimizes each pair of coupled regressions, which is based on a modification of the glm.nb method from the MASS package[1] in R. In particular, we attempt to find the roots of the partial derivative of $Q\left(\theta|\boldsymbol{X} = \boldsymbol{x},\tilde{\theta}\right)$ with respect to the shared δ_t and γ_t. Each of these derivatives however is simply the sum of the partial derivatives with respect to each parameter of the two components. Therefore, the standard procedure of fitting weighted negative binomial regressions can be reused whereby the part that finds the roots of the partial derivatives with respect to δ_t and γ_t, had they not been shared, is replaced by a routine that finds the roots of the sum of the partial derivatives across both components with respect to each parameter. On the other hand, the parts that find the roots of the partial derivatives with respect to α_t and β_t are the same as in the standard procedure for fitting weighted negative binomial regressions. The only other difference between our implementation and glm.nb is that ChromTime uses the HYBRD method from the MINPACK package[2] for finding roots of functions instead of Iterative Reweighted Least Squares (IRLS). In our tests, our optimization routine and glm.nb yielded very similar results for regular un-coupled weighted negative binomial regressions.

Optimizing the boundary movement components

The part of the expectation of the conditional complete log-likelihood that pertains to modelling the peak boundary movements is:

$$\sum_{i=1}^{M}\sum_{t=1}^{T-1}\sum_{s\in\{L,R\}}\sum_{d\in\left\{\substack{\text{EXPAND,}\\ \text{CONTRACT}}\right\}}\sum_{j=1}^{N_i} P\left(J_{i,s,t}=(-1)^{g(d)}j, D_{i,s,t}=d \middle| \mathbf{W}_i=\mathbf{w}_i;\tilde{\theta}\right)\log\left(\text{NB}(j-1;\mu_d,\delta_d)\right)$$

Again, this equation is equivalent to the equation for finding the maximum likelihood estimates of the coefficients and the dispersion parameter of one weighted negative binomial regression for each dynamic and time point that aims to predict the number of positions the left or the right boundary moves minus 1 ($j-1$, as response) from a single covariate which is the constant term equal to 1. The weights correspond to the posterior probability of moving the boundary by j positions, $P\left(J_{i,s,t}=(-1)^{g(d)}j, D_{i,s,t}=d \middle| \mathbf{W}_i=\mathbf{w}_i;\tilde{\theta}\right)$. The procedure to find the maximum likelihood estimates is the same as the one used in the previous section, except that no sharing of parameters is enforced between any of the negative binomial regressions.

Sharing dispersion parameter between negative binomial distributions

Sharing the dispersion parameter δ between two negative binomial distributions ensures that the distribution with the smaller mean value has higher probabilities compared to the distribution with the larger mean value for the lowest values of the support domain of the negative binomial distribution, and that the opposite holds for the largest values of the support domain. Here we will prove this claim. Let μ_1 and μ_2 be the means of two negative binomial distributions, *NB1* and *NB2*, respectively. Without loss of generality, we will assume that $0 \leq \mu_1 < \mu_2$. Dividing the probability mass functions of the two distributions gives:

$$\frac{NB1(k)}{NB2(k)} = \frac{\frac{\Gamma(k+\delta)}{k!\,\Gamma(\delta)}\left(\frac{\delta}{\mu_1+\delta}\right)^{\delta}\left(\frac{\mu_1}{\mu_1+\delta}\right)^{k}}{\frac{\Gamma(k+\delta)}{k!\,\Gamma(\delta)}\left(\frac{\delta}{\mu_2+\delta}\right)^{\delta}\left(\frac{\mu_2}{\mu_2+\delta}\right)^{k}} = \frac{\left(\frac{1}{\mu_1+\delta}\right)^{\delta}\left(\frac{\mu_1}{\mu_1+\delta}\right)^{k}}{\left(\frac{1}{\mu_2+\delta}\right)^{\delta}\left(\frac{\mu_2}{\mu_2+\delta}\right)^{k}} = \left(\frac{\mu_1}{\mu_2}\right)^{k}\left(\frac{\mu_2+\delta}{\mu_1+\delta}\right)^{\delta+k}$$

Since $\delta > 0$, substituting with k = *0*, gives:

$$\frac{NB1(0)}{NB2(0)} = \left(\frac{\mu_1}{\mu_2}\right)^{0}\left(\frac{\mu_2+\delta}{\mu_1+\delta}\right)^{\delta} = \left(\frac{\mu_2+\delta}{\mu_1+\delta}\right)^{\delta} > 1$$

Therefore, *NB1* has higher probability for k = *0* compared to *NB2*.

To prove that the opposite holds for the largest values of the support, we will take the limit of the above ratio for $k \to \infty$:

$$\lim_{k\to\infty}\frac{NB1(k)}{NB2(k)} = \lim_{k\to\infty}\left(\frac{\mu_1}{\mu_2}\right)^{k}\left(\frac{\mu_2+\delta}{\mu_1+\delta}\right)^{\delta+k} = \left(\frac{\mu_2+\delta}{\mu_1+\delta}\right)^{\delta}\lim_{k\to\infty}\left(\frac{\frac{\mu_1}{\mu_1+\delta}}{\frac{\mu_2}{\mu_2+\delta}}\right)^{k} = 0$$

The last equality holds, because:

$$\frac{\mu_1}{\mu_1+\delta} - \frac{\mu_2}{\mu_2+\delta} = \frac{\delta(\mu_1-\mu_2)}{(\mu_1+\delta)(\mu_2+\delta)} < 0 \;\Rightarrow\; \frac{\frac{\mu_1}{\mu_1+\delta}}{\frac{\mu_2}{\mu_2+\delta}} < 1$$

Therefore, for sufficiently large k *NB2* has higher probability compared to *NB1*.

We note that sharing the dispersion parameter for negative binomial mixture models is analogous to sharing the variance parameter in Gaussian mixture models.

Computing the most likely spatial dynamic and peak boundaries for each block across the whole time course

After the optimal values for all model parameters are estimated from the data, for each block the most likely positions of the peak boundaries at each time point are calculated. This procedure consists of two steps. First, ChromTime determines for each block all time points with significantly low probability of containing a false positive non-zero peak. Second, conditioned on those time points, ChromTime computes the most likely assignment of the peak boundary variables at each side and each time point.

During the first step, for each block and each time point ChromTime computes the posterior probability that the whole time point is modelled as background, $\varphi_{i,t} = \prod_{p=1}^{N_i} P(V_{i,t,p} = \text{BACKGROUND} | \mathbf{W}_i = \mathbf{w}_i)$. This probability can be interpreted as the probability of making a false positive non-zero length peak call as estimated by the model at time point *t* in block i. To determine significant time points with low false positive probability, $\varphi_{i,t}$, ChromTime computes a time point specific threshold, τ_t, at a predefined false discovery rate (0.05 by default) by applying the standard Benjamini-Hochberg procedure[3] on all values of $\varphi_{i,t}$ from time point *t*.

In the second step, for each block ChromTime computes the most likely sequence of assignments of the boundary positions, conditioned on the event that all time points that failed to pass the FDR threshold in the previous step for the block are assigned to having no peaks. In particular, ChromTime executes a dynamic programming algorithm similar to the Viterbi algorithm for hidden Markov models, which uses the following recursive formula to find the most likely position for the peak boundaries at each side, s (Left or Right) and enforces that time points that failed the FDR test contain no peaks:

$$\mathrm{DP}_{i,t,l,r} = \begin{cases} \log\left[P\left(\boldsymbol{O}_{i,1} = \boldsymbol{o}_{i,1}, Z_{i,1} = 1 \middle| B_{i,L,1} = l, B_{i,R,1} = r, \boldsymbol{X}_{i,1} = \boldsymbol{x}_{i,1}\right) P\left(B_{i,L,1} = l\right) P\left(B_{i,R,1} = r\right)\right] & ,t = 1 \\ \max\limits_{\substack{l_{t-1} \in [1, N_i+1] \\ r_{t-1} \in \begin{cases} [l_{t-1}-1, N_i] & \text{if } \varphi_{i,t} \le \tau_t \\ \{l_{t-1}-1\} & \text{otherwise} \end{cases}}} \begin{pmatrix} \mathrm{DP}_{i,t-1,l_{t-1},r_{t-1}} + \\ \log P\left(\boldsymbol{O}_{i,t} = \boldsymbol{o}_{i,t}, Z_{i,t} = 1 \middle| B_{i,L,t} = l, B_{i,R,t} = r, \boldsymbol{X}_{i,t} = \boldsymbol{x}_{i,t}\right) + \\ \log P\left(J_{i,L,t-1} = l_{t-1} - l \middle| D_{i,L,t-1} = d(l_{t-1} - l)\right) + \\ \log P\left(D_{i,L,t-1} = d(l_{t-1} - l)\right) + \\ \log P\left(J_{i,R,t-1} = r - r_{t-1} \middle| D_{i,R,t-1} = d(r - r_{t-1})\right) + \\ \log P\left(D_{i,R,t-1} = d(r - r_{t-1})\right) \end{pmatrix} & ,t > 1 \end{cases}$$

$$\text{where } d(j) = \begin{cases} \text{STEADY} & \text{if } j = 0 \\ \text{EXPAND} & \text{if } j \ge 1 \\ \text{CONTRACT} & \text{if } j \le -1 \end{cases}$$

and DP_i denotes the dynamic programing cube of size $T(N_i+1)^2$ that stores the log-likelihood for the best assignment of the peak boundary variables up to time point *t* in block *i*. Tracing the DP_i cube from the highest value on row *T* back to row 1 retrieves the best assignment of the peak end variables. Similarly to the expectation step of the EM phase, for blocks longer than MAX_BINS bins the best Viterbi path is chosen among the splits at the top MAX_BINS positions in the block sorted by their average foreground signal across all time points. Since MAX_BINS is a predefined constant, the whole procedure has the same time and space complexity as computing the expectations in the EM phase of ChromTime. The dynamic between any two time points is determined from the direction of the movement of the optimal positions of the corresponding boundaries.

Transcription factor binding and DNase I hypersensitivity data

In Figure 3A, TF binding data for GATA3 was used from the same study of mouse T cell development[4].

In Additional file 1: Figures S4Ai and S4Aiv, OCT4 and NANOG binding data for H1-hESC was downloaded from the ENCODE project [5]:

OCT4: https://www.encodeproject.org/files/ENCFF002CJF/@@download/ENCFF002CJF.bed.gz

NANOG: https://www.encodeproject.org/files/ENCFF002CJA/@@download/ENCFF002CJA.bed.gz

In Additional file 1: Figure S4Ai, P300 binding data for H1-hESC was downloaded from the ENCODE project [5]:

P300:

http://hgdownload.cse.ucsc.edu/goldenPath/hg19/encodeDCC/wgEncodeBroadHistone/wgEncodeBroadHistoneH1hescP300kat3bPk.broadPeak.gz

In Additional file 1: Figure S4Ai, IMR90 peaks for P300 generated from previously published data[6] were downloaded at 0.05 FDR from ChIP-Atlas[7]:
http://chip-atlas.org/view?id=SRX212184

Narrow peaks for all other TFs in Additional file 1: Figures S4Ai and S4Aii were downloaded from the ENCODE consortium[5] from the following URL:
http://hgdownload.cse.ucsc.edu/goldenPath/hg19/encodeDCC/wgEncodeAwgTfbsUniform/

In Additional file 1: Figures S4Ai and S4Aii, for DHSs we used DNaseI hypersensitivity peaks for H1 and IMR90 cells that were downloaded from the Roadmap Epigenomics Consortium[8]:
http://egg2.wustl.edu/roadmap/data/byFileType/peaks/consolidated/narrowPeak/E003-DNase.macs2.narrowPeak.gz
http://egg2.wustl.edu/roadmap/data/byFileType/peaks/consolidated/narrowPeak/E017-DNase.macs2.narrowPeak.gz

In Additional file 1: Figures S4Aiii, S4Biii and S7, ATAC-seq, HM, TF binding and gene expression data was used from a previously published study about stem cell reprogramming in mouse[9].

In Additional file 1: Figures S4Aiv and S4Biv aligned reads for DNaseI hypersensitivity were downloaded for H1 human embryonic stem cells, H1-derived neuronal progenitor cells and fetal brain tissue (epigenome ids: E003, E007, E082) from the Roadmap Epigenomics Consortium[8]:

http://egg2.wustl.edu/roadmap/data/byFileType/alignments/consolidated/E003-DNase.tagAlign.gz

http://egg2.wustl.edu/roadmap/data/byFileType/alignments/consolidated/E007-DNase.tagAlign.gz

http://egg2.wustl.edu/roadmap/data/byFileType/alignments/consolidated/E082-DNase.tagAlign.gz

Gene expression data in Additional file 1: Figure S4Biv was downloaded from the Roadmap Epigenomics Consortium[8]:

http://egg2.wustl.edu/roadmap/data/byDataType/rna/expression/57epigenomes.RPKM.pc.gz

Cell type specific and shared annotations were derived after subtracting one of the annotations from the other with the BedTools software[10] using the "*bedtools subtract –A"* command and by intersecting the two annotations with "*bedtools intersect"*, respectively.

Fold enrichments calculation

In Figure 3A and Additional file 1: Figure S4A, three types of ChromTime blocks (T1-Tn Steady, Tx-Tn Expand and T1-Tx Contract) were defined to start and end from the left most to the right most boundary, respectively, of the non-zero length peaks across all time points within each block. "T1-Tn Steady" blocks have non-zero length peaks at all time points with both left and right boundaries predicted as steady across all time points. "Tx-Tn Expand" blocks have a non-zero length peak at the last time point, have no contracting peak boundaries, and have at least one expanding peak boundary from a zero or non-zero length peak for at least one pair of consecutive time points. "T1-Tx Contract" blocks have a non-zero length peak at the first time point, have no expanding peak boundaries, and have at least one contracting peak boundary to a zero or non-zero length peak for at least one pair of consecutive time points.

In Additional file 1: Figure S3, ChromTime peaks annotated with each dynamic class (e.g. E/E, E/S, etc) at each pair of consecutive time points were defined to start and end from the left most to the right most coordinate, respectively, of the peaks at the corresponding pair of time points.

Fold enrichments of base pair overlap in Figures 3A, 6, and Additional file 1: Figures S3, S4A, and S7 were computed for each pair of genomic features by dividing the size of their observed overlap by the size of their expected overlap. For two genomic features *A* and *B*, the observed overlap was defined as the total number of bases in their intersection, $|A \cap B|$. The expected overlap was defined based on a binomial null model that preserves the size of each feature and treats the two features as independently distributed within a certain set of eligible background genomic positions, *G*:

$$\mathrm{E}(A, B) = |A| * \frac{|B|}{|G|}$$

where |*G*| denotes the size of the set of all eligible positions. In Figure 3A and Additional file 1: Figure S4A the set of eligible background positions was defined for each time course as all genomic bases covered by ChromTime peaks for that time course. In Additional file 1: Figure S3, the set of eligible background positions at each time point was defined as all bases in the union of all predicted ChromTime peaks from both replicates at that time point. In Figure 6 and Additional file 1: Figure S7, the set of eligible background positions is defined to be all genomic bases in the corresponding genome. In addition, in Figure 6 and Additional file 1: Figures S3 and S7 in order to avoid extremely high enrichments due to very rare predicted dynamics classes a pseudo-count of 200 bp (i.e. one genomic bin) was added to each overlap. In Additional file 1: Figure S3, the fold enrichments for each pair of consecutive time points were $\log_2$-transformed and the average of the log-transformed values across all time point pairs is shown. In Figure 6 and Additional file 1: Figure S7, geometric means were taken across enrichments at each pair of consecutive time points and the resulted average enrichments were capped at a maximum value of 50. Clustering with optimal leaf ordering[11] was performed in Figure 6 and Additional file 1: Figure S7 after this procedure.

Gene expression data processing

Gene expression data used in Figures 3B, 4, 5, and Additional file 1: Figures S4B, S5, and S6 was used from the same studies that provided the corresponding ChIP-seq, ATAC-seq, or DNase-seq data. Prior to all analysis, the gene expression values (RPKM) were transformed with $\log_2(1+\text{RPKM})$. The $\log_2$-transformed values were then normalized to have mean of 0 and variance of 1 within each time point for each dataset. In Figure 3B and Additional file 1: Figure S4B, we used all blocks with at least one non-zero length peak called with ChromTime at any time point in the time course. Peaks that did not overlap a TSS were excluded from this analysis. For peaks that overlap multiple TSSs, the average gene expression change across all overlapping TSSs was used.

Cumulative average gene expression change as function of peak boundary ranks when boundaries are sorted by ChromTime posterior probabilities

In Figure 3B and Additional file 1: Figure S4B, we used all blocks with at least one predicted non-zero length peak that overlaps annotated TSSs. For each such block, i, and each pair of consecutive time points, t and $t+1$, we associated both the left and the right peak boundaries, $b_{i,L}$ and $b_{i,R}$ with the average gene expression difference between those time points, $\delta_{i,t}$, across all genes whose TSSs overlap the block. Then, we sorted the boundaries in decreasing order by their ChromTime posterior probability for EXPAND dynamic (left plots) and CONTRACT dynamic (right plots) at each pair of consecutive time points. Next, for each dynamic d (EXPAND or CONTRACT) and each posterior rank, k, we computed the cumulative average gene expression difference between time points t and $t+1$ across all peak boundaries with equal or better posterior rank as

$$\bar{\delta}_{d,k,t} = \frac{1}{k}\sum_{r=1}^{r=k} \delta_{\mathscr{b}(d,r,t),t}$$

where $\mathscr{b}(d,r,t)$ denotes the block index corresponding to the boundary with rank r when peak boundaries are sorted by their posterior probabilities of dynamic d at time point t. The plots in both figures show $\bar{\delta}_{d,k,t}$ values (Y-axis) as function of rank k (X-axis).

Average rank differences based on gene expression change

In Figure 4 and Additional file 1: Figure S5 we compared whether ranking peak boundaries by ChromTime posterior probabilities for EXPAND (left panels) and CONTRACT (right panels) dynamics has better agreement with gene expression changes than ranking peak boundaries by the number of genomic bases they move between consecutive time points when peaks are called at each time point by using input data only from that time point. The above comparison provides insights into whether ChromTime's posterior probabilities, which are computed by reasoning jointly about all time points in the time course, have benefits compared to analyzing boundary movements of peaks that are called at each time point in isolation. To compute the latter in Figure 4 and Additional file 1: Figure S5A we applied ChromTime to call peaks at each time point by using as input only data from that time point (ChromTime SINGLE). We then used the boundaries of those peaks that overlapped predicted ChromTime peaks called by using data from all time points in the time course (ChromTime ALL). In Additional file 1: Figure S5B, we used the boundaries of broad peaks called with MACS2[12] with the --broad option that overlap blocks with predicted ChromTime ALL peaks. To call MACS2 peaks in H3K4me2 in mouse T cell development[4] we additionally used "--nomodel --extsize 200" options. In Additional file 1: Figure S5C, we used the boundaries of peaks called with SICER[13] with parameters as recommended[14] that overlap blocks with predicted ChromTime ALL peaks.

To determine whether ranking based on ChromTime posteriors or ranking based on boundary movements between consecutive time points is better, we evaluated the consistencies of these rankings with respect to ranking all boundaries by the change in gene expression of genes whose TSS directly overlaps predicted peaks. Blocks with peaks that did not overlap a TSS were excluded from the analyses performed for Figure 4 and Additional file 1: Figure S5. For blocks with peaks that overlap multiple TSSs, the average gene expression change across all overlapping TSSs was used.

Between a pair of consecutive time points each block is associated with the movements of two boundaries – one boundary on the left side and one on the right side. If M is the total number of blocks with predicted peaks from both methods in each pairwise comparison, then the total number of

boundaries at any given time point is $2M$. These boundaries include both the boundaries of predicted zero length and non-zero length peaks. For a pair of consecutive time points, t and $t+1$, each of these boundaries can either stay steady, or expand or contract relative to time point t.

Expanding peak boundaries of H3K4me2 and H3K4me3 peaks are expected to be found near genes that increase in gene expression at time point $t+1$ relative to time point t, and vice versa for contracting boundaries. To quantify the degree to which ranking by posteriors and ranking by boundary movements of peaks in isolation is more consistent with gene expression changes, for each pair of consecutive time points t and $t+1$ we computed the following quantities for each boundary i and dynamic *D*:

1) $\mathrm{CT}(i, D)$ – rank of boundary i when boundaries are sorted by ChromTime posteriors of dynamic *D* (where *D* is one of EXPAND (left plots) or CONTRACT (right plots)) in descending order from the highest to the lowest posterior.
2) $\mathrm{BM}(i, D)$ – rank of boundary i when boundaries are sorted by the number of genomic bases that the boundaries of the overlapping peaks called in isolation move. For *D* = EXPAND (left plots), this ranking is performed in descending order from the most expanding to the most contracting boundary, and vice versa for *D* = CONTRACT (right plots). The number of genomic bases that a boundary moves is calculated as $(-1)^{\notin(s)}(b_{i,s,t+1} - b_{i,s,t})$, where $b_{i,s,t+1}$ and $b_{i,s,t}$ are the genomic positions of the peak boundary i on side s (left or right) at times $t+1$ and t respectively, and $\notin(s) = 1$ for left end boundaries and $\notin(s) = 0$ for right end boundaries. To handle cases, where ChromTime SINGLE or MACS2 or SICER did not call peaks at some time points within a block, zero length peaks were created artificially, so that the appearance of non-zero length peaks at time points after the first one is treated as a positive boundary movement from a zero length peak in the center of the new peak and the disappearance of non-zero length peaks is treated as a negative boundary movement to a zero length peak in the center of the disappeared peak. Also, if two consecutive time points have no peaks, then the boundary movements for both the left and right boundaries are set to 0. This procedure ensures that for each ChromTime ALL peak

boundary movement, there exists a corresponding boundary movement based on peaks called in isolation.

3) $\Delta E(i, D)$ – rank of boundary i when boundaries are sorted by the change in gene expression at time point $t+1$ relative to time point t of the overlapping TSS. The gene expression change at each TSS is quantified as $E_{t+1} - E_t$, where and E_{t+1} and E_t are the normalized gene expression levels at time points $t+1$ and t, respectively. For *D*=EXPAND (left plots), this ranking is performed in descending order (i.e. most up-regulated genes rank first and most down-regulated genes rank last), and vice versa for *D*=CONTRACT (right plots).

In all rankings, ties were broken randomly. Then for each rank k in rankings *CT* and *BM*, where $1 \le k \le 2M$, and dynamic *D* we computed the average $\Delta E(i, D)$ rank of all boundaries up to and including rank *k*:

$$\mu_{CT}(k, D) = \frac{1}{k} \sum_{k\prime=1}^{k} \Delta E(CT^{-1}(k', D), D)$$

and

$$\mu_{BM}(k, D) = \frac{1}{k} \sum_{k\prime=1}^{k} \Delta E(BM^{-1}(k', D), D)$$

where $CT^{-1}(k', D)$ and $BM^{-1}(k', D)$ denote the inverse functions of the rankings CT and BM, respectively, which return the boundary of rank k' according to the corresponding ranking. The two quantities, $\mu_{CT}(k, D)$ and $\mu_{BM}(k, D)$, measure the degree to which rankings CT and BM associate with differential gene expression as measured by the ranking ΔE up to the first k boundaries ordered by each ranking. In particular, $\mu_{CT}(k, D) < \mu_{BM}(k, D)$ corresponds to the case where CT is more consistent with ΔE than BM is, because the first k boundaries according to CT on average rank higher in terms of gene expression changes compared to the first k boundaries according to BM, and vice versa for $\mu_{CT}(k, D) > \mu_{BM}(k, D)$. In Figures 4Aii, 4Bii and the top row in Additional file 1: Figures S5A-C, for each pair of consecutive time points t and $t+1$ we plot the difference $\delta(k, D) = \mu_{BM}(k, D) - \mu_{CT}(k, D)$ as a function of k. Thus, positive values correspond to ranks for which CT better associates with gene expression as measured by ΔE than BM, and vice versa for negative values. The shaded regions correspond to 95% confidence intervals.

Finally, due to large fluctuations of the μ_{CT} and μ_{BM} quantities in the top ranks, the plots are shown for $k \geq 20$.

Gene expression changes as function of signal changes for different predicted ChromTime dynamics

In Figure 5 and Additional file 1: Figure S6 gene expression changes are plotted as function of signal density change and method-specific differential peak score respectively for all peaks annotated with the same predicted ChromTime dynamics. First, each block i was associated with the difference of the normalized $\log_2$ gene expression (as defined in the **Gene expression** section) at the nearest TSS within 50kb at each pair of consecutive time points t and $t+1$, $\Delta e_{i,t}$.

In Figure 5, to compute the change of signal density we first computed the $\log_2$ Control-normalized signal density for each block i at each time point t, as:

$$D_{i,t} = \log_2(1 + RPKM_{i,t}) - \log_2(1 + \lambda_{i,t})$$

where $RPKM_{i,t}$ denotes the number of reads per kilobase per million mapped reads (RPKM) for the corresponding chromatin mark, and $\lambda_{i,t}$ denotes the average expected number of reads for all bins in block i at time point t. The RPKM and $\lambda_{i,t}$ values for each block at each time point were computed over the same genomic territory spanning from the left most to the right most coordinate of the predicted peaks across all time points in the block. To compute the change in signal density between consecutive time points, we computed the difference between the corresponding $D_{i,t}$ values:

$$\delta_{i,t} = D_{i,t+1} - D_{i,t}$$

To visualize the relationship between signal density changes and gene expression changes, the tuples $(\delta_{i,t}, \Delta e_{i,t})$ were pooled together across all time points in each dataset and a Loess regression with linear

polynomials was fitted with the loess function[15] in R with default parameters except for degree=1. In cases with too many tuples the R package required excessive memory to compute the loess curves and, thus, 10,000 tuples were chosen at random as input for the loess function.

In Additional file 1: Figure S6, the same procedure was applied except that $\delta_{i,t}$ values were defined as method-specific differential peak scores of peaks called by different methods. Two independent differential peak callers were used, MACS2[12] and SICER[13], which were recommended by a previous study that evaluated a number of differential peak callers[14]. For MACS2, we used ”--nomodel --extsize 147” options. For SICER, we used FDR threshold of 0.01, window of 200 and gap of 600. With each differential caller we called differential and common peaks between every pair of consecutive time points in each time course according to the instructions in the evaluation study[14]. For each peak caller, we intersected ChromTime blocks with all called peaks from the caller and performed the analysis only on peaks identified by both ChromTime and the corresponding differential peak caller. We kept overlapping continuous segments only if the overlapping differential peaks from MACS2 or SICER were called at pairs of time points, which were between the first and the last time point with non-zero length peaks in ChromTime blocks. In cases where ChromTime peaks overlapped multiple MACS2 or SICER differential peaks, all overlaps were used in Additional file 1: Figure S6.

For MACS2, the differential score was defined as the $\log_2$ fold change of the signal of each differential or common peak as computed by MACS2.

For SICER, the differential score for each peak was defined as:

$$(-1)^q(-\log_{10}\min(\mathrm{FDR\_A\_vs\_B}, \mathrm{FDR\_B\_vs\_A}))$$

where

$$q = \begin{cases} 1 & \text{if FDR\_A\_vs\_B } < \text{FDR\_B\_vs\_A} \\ 0 & \text{otherwise} \end{cases}$$

The differential score for SICER has a negative sign for SICER peaks with enriched signal at the previous time point compared to the next time point, and a positive sign for peaks with enriched signal at the next time point compared to the previous time point. For peaks for which the FDR outputted by SICER was 0, the differential score was defined as the maximum absolute value of the differential scores across all peaks with non-zero FDRs multiplied by $(-1)^q$ in order to take into account the direction of enrichment.

Analysis of directional preferences of spatial dynamics of chromatin marks

The average log-ratios in Figure 7 were computed across all tested datasets for the corresponding chromatin marks (**Table 1**). For each time course, we split all ChromTime peaks into two groups, TSS+-1kb and TSS distal. The TSS+-1kb group contains all peaks whose distance to the nearest TSS is less than 1kb as measured with the "bedtools closest" software[10]. All other peaks were put in the TSS distal group. For each dataset and each group, we computed the log ratios for each pair of consecutive time points after adding a pseudo-count of 10 to each group. We then averaged those log-ratios across all time points in the dataset. For marks mapped in at least six time courses, we then plotted the average across all tested datasets as a solid black line in each subplot. A two-tailed Mann-Whitney test was performed for these marks to assess the statistical significance of the difference between the TSS+-1kb and TSS distal groups with the SciPy library[16]. To compute averages and test statistics in Figure 7 and Additional file 1: Figure S8, all datasets were treated as independent, except in the case of the mouse hematopoiesis[17] and human hematopoiesis[18] data. For human hematopoiesis, data was pooled from all donors in the study. For both the human and mouse hematopoiesis time courses, we applied ChromTime on data from each branch of the corresponding hematopoietic tree and computed a single average across all branches for the corresponding enrichment. The single average was then used in place of the values for each individual branch. This was done in order to avoid biasing statistics towards the mouse or human hematopoiesis data, since branches in the hematopoietic trees overlap substantially and, thus, cannot be treated as independent datasets.

References

1. Venables WN, Ripley BD. Modern Applied Statistics with S. Fourth. New York: Springer; 2002. http://www.stats.ox.ac.uk/pub/MASS4.

2. Moré, Garbow B, Hillstrom K. User Guide for MINPACK-1. ANL-80-74, Argonne Natl Lab. 1980. http://www.mcs.anl.gov/~more/ANL8074a.pdf. Accessed 25 Jun 2018.

3. Benjamini Y, Hochberg Y. Controlling the False Discovery Rate: A Practical and Powerful Approach to Multiple Testing. Journal of the Royal Statistical Society. Series B (Methodological). 1995;57:289–300. doi:10.2307/2346101.

4. Zhang JA, Mortazavi A, Williams BA, Wold BJ, Rothenberg E V. Dynamic transformations of genome-wide epigenetic marking and transcriptional control establish T cell identity. Cell. 2012;149:467–82. doi:10.1016/j.cell.2012.01.056.

5. ENCODE Project Consortium. An integrated encyclopedia of DNA elements in the human genome. Nature. 2012;489:57–74. doi:10.1038/nature11247.

6. Jin F, Li Y, Dixon JR, Selvaraj S, Ye Z, Lee AY, et al. A high-resolution map of the three-dimensional chromatin interactome in human cells. Nature. 2013;503:290–4. doi:10.1038/nature12644.

7. Oki S, Ohta T, Shioi G, Hatanaka H, Ogasawara O, Okuda Y, et al. Integrative analysis of transcription factor occupancy at enhancers and disease risk loci in noncoding genomic regions. bioRxiv. 2018;:262899. doi:10.1101/262899.

8. Roadmap Epigenomics Consortium, Kundaje A, Meuleman W, Ernst J, Bilenky M, Yen A, et al. Integrative analysis of 111 reference human epigenomes. Nature. 2015;518:317–30. doi:10.1038/nature14248.

9. Chronis C, Fiziev P, Papp B, Butz S, Bonora G, Sabri S, et al. Cooperative Binding of Transcription Factors Orchestrates Reprogramming. Cell. 2017;168:442–459.e20. doi:10.1016/j.cell.2016.12.016.

10. Quinlan AR, Hall IM. BEDTools: a flexible suite of utilities for comparing genomic features. Bioinformatics. 2010;26:841–2. doi:10.1093/bioinformatics/btq033.

11. Bar-Joseph Z, Gifford DK, Jaakkola TS. Fast optimal leaf ordering for hierarchical clustering. Bioinformatics. 2001;17 SUPPL. 1:S22-9. doi:10.1093/bioinformatics/17.suppl_1.S22.

12. Zhang Y, Liu T, Meyer CA, Eeckhoute J, Johnson DS, Bernstein BE, et al. Model-based analysis of

ChIP-Seq (MACS). Genome Biol. 2008;9:R137. doi:10.1186/gb-2008-9-9-r137.

13. Xu S, Grullon S, Ge K, Peng W. Spatial clustering for identification of ChIP-enriched regions (SICER) to map regions of histone methylation patterns in embryonic stem cells. Methods Mol Biol. 2014;1150:97–111. doi:10.1007/978-1-4939-0512-6_5.

14. Steinhauser S, Kurzawa N, Eils R, Herrmann C. A comprehensive comparison of tools for differential ChIP-seq analysis. Brief Bioinform. 2016;17:953–66. doi:10.1093/bib/bbv110.

15. W. S. Cleveland EG and WMS. Local regression models. 1992.

16. Jones E, Oliphant T, Peterson P. SciPy: Open source scientific tools for Python. 2001. http://www.scipy.org/. Accessed 26 Nov 2017.

17. Lara-Astiaso D, Weiner A, Lorenzo-Vivas E, Zaretsky I, Jaitin DA, David E, et al. Chromatin state dynamics during blood formation. Science (80-). 2014;345:943–9. doi:10.1126/science.1256271.

18. Corces MR, Buenrostro JD, Wu B, Greenside PG, Chan SM, Koenig JL, et al. Lineage-specific and single-cell chromatin accessibility charts human hematopoiesis and leukemia evolution. Nat Genet. 2016;48:1193–203. doi:10.1038/ng.3646.
